# The multicomponent residue depletion of *Gelsemium elegans* in pig tissues, urine, and plasma

**DOI:** 10.3389/fvets.2022.1111782

**Published:** 2023-01-13

**Authors:** Yong Wu, Xue-Ming Long, Gao-Feng Liu, Xia Bai, Zhi-Liang Sun, Zhao-Ying Liu

**Affiliations:** ^1^College of Veterinary Medicine, Hunan Agricultural University, Changsha, Hunan, China; ^2^Hunan Provincial Institute of Veterinary Drugs and Feed Control, Changsha, Hunan, China; ^3^Hunan Canzoho Biological Technology Co., Ltd., Liuyang, Hunan, China

**Keywords:** *Gelsemium*, LC-MS/MS, residue depletion, gelsedine, target tissue

## Abstract

**Introduction:**

*Gelsemium elegans* (*G. elegans*) as a traditional medicinal plant used in livestock production. The use of *G. elegans* in veterinary clinics may pose safety risks to human health.

**Objectives:**

The aim of this study was to investigate tissue residue depletion in pigs fed *G. elegans* powder.

**Methods:**

A precise quantitation method and a simultaneous semi-quantitation method for multiple components independently of standards in pig tissues were developed for the first time. The two methods were validated in terms of specificity, LODs, LOQs, linearity, accuracy, precision, and matrix effects. They were then applied to a tissue residue depletion study after *G. elegans* powder at a dose of 2% per kg feed were fed to pigs.

**Results:**

Compared with precise quantitation, the method validation results indicated that the semi-quantitation method was reliable and acceptable for multicomponent quantification independent of standards. Many *G. elegans* alkaloids are widely distributed in most tissues of pigs. Tissue residue depletion studies indicated that 14-hydroxygelsenicine, 11-hydroxygelsenicine, and gelsemoxonine could be used as potential residue markers, and pancreas, small intestine, and lung tissues could be considered as potential residue target tissues of *G. elegans*. In addition, both urine and plasma could be used to predict 14-hydroxygelsenicine and gelsemoxonine residues in the liver, pancreas, and small intestinal tissues of pigs.

**Conclusion:**

The developed semi-quantification method can be applied to monitor the application and residue of *G. elegans*. The results provide scientific evidence for evaluating the safety of animal-derived food from *G. elegans* for consumers and will be helpful for its application and future development.

## Introduction

Phytogenic feed additives (PFAs) are plant-derived products often used in animal feed to improve the performance of livestock in agriculture (1, 2). PFAs are mainly used in livestock production in China due to their clinical effects, such as antioxidant (3–6) and antibacterial activities (1, 2, 7, 8), and promotion of intestinal activity promotion (9–11) efficacy. The potential issue of PFAs residues, particularly those that contain toxic components, has garnered widespread attention in recent years. However, there is still little systematic and scientific research on the potential risk that the residues of PFAs pose to the safety of food production. Toxic residues in PFAs can enter the human body through the consumption of animal-derived food products or ecological chains, causing potential safety risks to human health.

*Gelsemium elegans* Benth. (*G. elegans*) is an evergreen woody vine of the genus *Gelsemium* of the family Loganiaceae, distributed in downstream Yangtze River areas such as Guangxi, Guangdong, and Fujian (12–14). As a traditional Chinese medicine, *G. elegans* has pharmacological therapeutic effects on rheumatoid and neuropathic pain (15). The narrow therapeutic window limits its clinical use in human medicine. Some studies have attempted to widen the therapeutic window by demonstrating a positive mechanism in cancer treatment. In recent years, many researchers have studied the effects of dietary *G. elegans* on pigs (16, 17), goats (18), and fish (19). Results showed G. elegans could promote the growth of livestock. It was also recorded in the ancient compendium of materia medica and the Chinese veterinary pharmacopeia (2020 edition). Currently, *G. elegans* is used as PFA in veterinary clinics in China. *Gelsemium elegans* is well known for its extreme toxicity. Recently, there have been reports of poisoning and death in China due to the inadvertent consumption of *G. elegans* by the mistaken identification of this toxic plant (20, 21). The use of *G. elegans* in veterinary clinics may pose safety risks to human and animal health. Thus, it is necessary to investigate the tissue residue of *G. elegans* in food animals, which is conducive to the protection of food safety and human health.

The composition of *G. elegans* is highly complex. To date, 121 alkaloids have been isolated and identified from *G. elegans* plants, among which indole alkaloids are considered the main active components (13, 14, 22, 23). The different chemical structures of indole alkaloids have led to their division into six categories: sarpagine-type, koumine-type, gelsemine-type, gelsedine-type, humantenine-type, and yohimbane-type. Gelsemine (belonging to the gelsemine-type group), koumine (belonging to the koumine-type group), and gelsenicine (belonging to the gelsedine-type group) are the major components of *G. elegans* (14, 24). Among them, the relative abundance of gelsemine was the highest, at ~0.67%, koumine was in the middle, and the content of gelsenicine was the lowest and exhibited the highest toxicity. According to the drug-time curves of these three alkaloids, their absorption was rapid in rats, but certain differences in their pharmacokinetic characteristics persisted (25–27). Only one study has reported the tissue distribution of gelsemine in rats, whereby gelsemine was rapidly distributed to the stomach, spleen, kidneys, brain, liver, heart, and lungs after intravenous administration. Furthermore, the residual amount of gelsemine in the stomach and spleen was high, while that in the brain and liver was low (25). However, it is worth noting that although significant progress has been made in understanding the tissue distribution of gelsemine and the pharmacokinetic characteristics of alkaloids, current research on *G. elegans* alkaloid residue depletion in animal tissues is far from satisfactory. A single constituent or few active ingredients cannot reflect the collective active ingredients, characteristics, or efficacy of *G. elegans*. This consensus was not only accepted by an increasing number of pharmacists but was also in line with the essential characteristics of a holistic approach to treatment.

The purpose of this study was to establish a precise quantitation method and a simultaneous semi-quantitation method for multiple components independently of standards in pig tissues by UPLC-MS/MS. The two methods were then applied for the residue depletion of several *G. elegans* alkaloids in pig tissues, urine, and plasma. Finally, the correlation between potential residue markers among tissues and biological fluids was investigated. The results of this study provide a theoretical basis for the clinical application of *G. elegans* and to ensure food safety and human health.

## 2. Experimental

### 2.1. Materials and reagents

Gelsemine (purity ≥ 98%), koumine (purity ≥ 98%), and gelsenicine (purity ≥ 95%) were purchased from Shanghai Kang Biao Chemicals Co. LTD (Minhang District, Shanghai, China). Chromatography-grade methanol, formic acid, and acetonitrile were obtained from Merck Chemicals Co. (Darmstadt, Germany). Deionized water was prepared using a Milli-Q water purification system (Millipore, Billerica, MA, USA). All the other chemicals and reagents used in the experiments were of analytical grade.

### 2.2. Collection and pretreatment of *G. elegans*

In total, 50 kg of whole *G. elegans* plant was collected from the Fujian Province, China. The samples were authenticated by Associated Professor Qi Tang at the Hunan Agricultural University. Crude *G. elegans* samples were dried and milled into powders. This powder was then passed through a 100-mesh sieve before mixing with the feed. The extraction and purification of *Gelsemium* total alkaloids (content over 80%) have been described in our previous study (28, 29).

### 2.3. Sample preparation

Blank tissue samples (muscle, liver, and kidneys of pigs) were purchased from local markets (Changsha, China) to obtain free *Gelsemium* alkaloid tissues, which were used for the two methodology validations. The tissues were homogenized in a high-speed grinder and stored at −80°C.

After thawing and returning to room temperature, samples (2.00 ± 0.02 g) were accurately weighed into 50.0 mL centrifuge tubes. Acetonitrile (10.0 mL) was added and immediately vortexed for 60 s. Then, 100.0 μL formic acid was added and vortexed again, and the mixture was extracted at 40°C by ultrasonication for 30 min. The supernatant was transferred to a fresh centrifuge tube and centrifuged at 8,000 rpm for 5 min. The residual samples were re-extracted in the same way, and the supernatant was combined. Then, 10.0 mL of the supernatant was evaporated to dryness using nitrogen at 35°C. About 1.0 mL of methanol/0.1% formic acid-water (10:90, v/v) and N-hexane (0.5 mL) were added to the tube, and the mixture was vortexed for 30 s. The solution was then centrifuged at 10,000 rpm for 5 min. The lower layer was extracted with a 1.0 mL syringe and through a 0.22 μm microporous membrane. Subsequently, 5.0 μL of the filtered solution was injected into the UPLC-MS/MS system for the analysis.

### 2.4. Preparation of standard solutions and matrix-matched standard curves

For precise quantitation, standard stock solutions (100.0 μg/L) of gelsemine, koumine, and gelsenicine were prepared separately in pure methanol. Working standard solutions containing all three alkaloids were diluted with methanol/0.10% formic acid-water (10:90, v/v) to a concentration range of 0.5–200 μg/L. For the semi-quantitation assay, Gelsemium total alkaloid solution (1,000.0 μg/L) was prepared separately in pure methanol and stored at −80°C in the absence of light. Working standard solutions of Gelsemium total alkaloids were freshly prepared at the desired concentrations through serial dilution of the stock solutions with methanol/0.10% formic acid-water (10:90, v/v).

For the matrix-matched standard curves, blank tissue was extracted as described above to obtain blank matrix solutions. Matrix-matched standard solutions were then obtained by consecutively diluting the stock solution of standard or Gelsemium total alkaloids with blank matrix solutions to the desired concentrations.

### 2.5. UPLC-MS/MS conditions

UPLC-MS/MS analysis was performed using a Shimadzu chromatography-30A system (Shimadzu Co., Kyoto, Japan) and an AB Sciex QTRAP 4500 mass spectrometer (AB SCIEX, Framingham, USA). The UPLC system was equipped with a DUG-30A5 degasser, LC-30AD binary pump, CTO-30A column controller, and SIL-30AC autosampler. Separation was achieved on a Waters ACQUITY BEH C18 column (2.1 mm × 100 mm, 1.7 μm) at a constant flow rate of 0.3 mL/min at 35°C. Desirable chromatographic separation of alkaloids was achieved using mobile phase A (0.1% formic acid-water) and mobile phase B (acetonitrile). The gradient elution program for the precise quantitation of the three alkaloids (gelsemine, koumine and gelsenicine) was as follows: 0–2.0 min, 10% B; 2.0–6.0 min, 65% B; 6.0–8.0 min, 65% B; and 8.0–10.0 min, 90% B. The sample injection volume was 20.0 μL. The gradient elution program for *Gelsemium* total alkaloids (26 alkaloids) was as follows: 0–1.5 min, 8% B; 1.5–10 min, 40% B; 10.1–12.0 min, 95% B; and 12.1–14.0 min, 8% B.

Mass spectrometric detection was performed in the positive electrospray ionization (ESI) mode with multiple reaction monitoring (MRM) or scheduled derived multiple reaction monitoring (sMRM). Instrument control, data acquisition, and analysis were performed using Analyst software (version 1.6.3). The detailed MS/MS analytical conditions are described in our previously published methods for plasma (29, 30).

### 2.6. Method validation

In the present study, the two methods were validated in terms of specificity, LODs, LOQs, linearity, accuracy, precision, and matrix effects. The precise quantitation UPLC-MS/MS method of the three standard compounds was developed first. Owing to the lack of authentic standards for many components in *G. elegans*, another simultaneous semi-quantitation of multiple components independent of standards was validated. The method was then compared with precise quantitation to demonstrate that the semi-quantitation method was reliable and acceptable for multicomponent quantification. The two methods were determined by spiking blank tissues (muscle, liver, and kidneys) with three alkaloid standard solutions and *Gelsemium* total alkaloid standard solutions.

The specificities of the two methods were evaluated based on the presence of interfering substances around the alkaloid retention times by comparing the MRM ion chromatograms of blank tissues to those of the corresponding spiked tissue samples containing the standards. LOD values were defined as three times the signal-to-noise ratio (*S/N*) and established by the following steps. First, 20 blank samples of pig tissues were analyzed, and the *S/N* was calculated within the retention time window in which each analyte was expected to elute. LOQ values were defined as alkaloid responses that yielded *S/N* values > 10.

Matrix-matched standard curves were constructed to 6–8 levels. The calibration curves were obtained using the weighted (1/x^2^) least squares linear regression method by measuring the peak area of each alkaloid. The linear regression equation for each component is expressed as y = ax + b, where y represents the peak area of the target analyte and x represents the concentration of each alkaloid. For the semi-quantitation method, the concentration of each alkaloid was not the actual concentration, but that of the *Gelsemium* total alkaloids.

The inter-day and intra-day accuracy and precision batches comprised six replicates of each tissue at three different concentration ranges (low: 5.0, 10.0, 20.0, and 50 μg/kg; medium: 50.0, and 100 μg/kg; high: 100.0 and 150 μg/kg) and were calculated by the ratio of the measured concentration to the theoretical concentration, and the relative standard deviation (RSD) between the samples was determined. The matrix effect (ME) on the ionization of the analytes were evaluated by comparing the peak areas of the analytes in the standard solution with those in the matrix extract solution.

### 2.7. Residue depletion study

Forty 2-month-old healthy male (20 ± 2 kg) swine were purchased from the Xinwufeng Limited Company (Changsha, China). All swine were observed for 1 week before the experiment. Food intake, water intake, feces, and temperature were recorded daily. All swine were medically examined by experienced veterinarians to ensure their health. The animals were randomly assigned to two groups, A (10 pigs) and B (30 pigs), in which group A was fed a basic ration without the tested drug, and group B was administered *G. elegans* powder at a dose of 2% per kg feed. The temperature was maintained at ~25°C.

In this experiment, the treatment lasted for 45 d. One pig in group A and five pigs in group B were sacrificed at 0.25, 1, 3, 7, and 15 d after the last dose. At each slaughter time point, blood samples were collected in 5.0 mL sodium heparin anticoagulation tubes and then centrifuged at 3,500 rpm for 15 min, and the supernatant plasma was transferred to 2.0 mL centrifuge tubes. Immediately, tissues (including muscle, liver, kidneys, heart, spleen, lungs, brain, adrenal gland, spinal cord, pancreas, small intestine, plasma, and gonads) were collected and placed in plastic bags in an ice bath. Urine samples were collected from bladders. All samples were stored at −80°C until analysis. All trials were conducted following the Guide for the Care and Use of Laboratory Animals and approved and were supervised by the Animal Care and Use Committee, Hunan Agricultural University.

## 3. Results

### 3.1. Optimization of sample preparation

In the residue analysis of PFAs in tissues, a key point is the sample extraction and cleanup step required for the component of interest from biological matrices. In the present study, pretreatment methods involved investigating sample extraction using three standard alkaloids (gelsemine, koumine, and gelsenicine). To better understand the effects of different ratios of mixed organic solvents and aqueous solvents on the extraction efficiency of the tested analytes, 10 mL of mixed organic-aqueous solvents at different ratios was tested at a spiking concentration of 100.0 μg/kg of gelsemine, koumine, and gelsenicine. The acids were then fortified in aqueous solvents. The results demonstrated that recoveries of more than 80% were achieved with acidic acetonitrile with an aqueous solvent ratio of < 5%. Therefore, acidified acetonitrile was used as the extraction reagent. However, the 1% formic acid-acetonitrile extraction reagent quickly caused the matrix to aggregate, resulting in dispersion difficulty and a reduced extraction recovery rate. Thus, in this study, acetonitrile was first utilized for extraction and formic acid was added for acidification.

### 3.2. Method validation of the two proposed methods

Until now, there has been no multicomponent residue analysis of *G. elegans* owing to the lack of powerful simultaneous quantitative methods and the difficulty of accurate quantification without pure standard products. The two proposed methods were validated in terms of specificity, LODs, LOQs, linearity, accuracy, precision, and matrix effects. The precise quantitation parameters of three characteristic compounds (gelsemine, koumine, and gelsenicine) were compared with those of 22 component semi-quantitation.

As shown in Supplementary Figure 1, there were no interfering peaks with those of the three alkaloids in the blank pig tissue sample obtained by the precise quantitation method. Moreover, as shown in Supplementary Figure 2, there were no interfering peaks with those of the 22 target analytes in the blank pig muscle sample obtained using the semiquantitative approach. Typical chromatograms of blank liver and kidney tissues and blank tissue samples spiked with *Gelsemium* total alkaloids are shown in Supplementary Figures 3, 4. The results showed that the two methods had good specificity.

Calibration curves for the semiquantitative analysis were constructed using *Gelsemium* total alkaloids, whereas those for precise quantitation were obtained from chemical standards. As presented in Supplementary Table 1, semi-quantitation yielded satisfactory correlation coefficients (*r* > 0.9923) in the **three** tissue matrices (muscle, liver, and kidneys) and a linear concentration range for the three compounds, indicating its reliability and comparable performance to that of precise quantitative analyses. Table 1 summarizes the LOD and LOQ results for all the analyzed alkaloids.

**Table 1:** LODs and LOQs for *G. elegans* alkaloids in different matrices (muscle, liver, and kidney).

**Name**	**Analytes**	**Muscle (**μ**g/kg)**		**Liver (**μ**g/kg)**		**Kidney (**μ**g/kg)**	
		**LOD**	**LOQ**	**LOD**	**LOQ**	**LOD**	**LOQ**
Koumidine	GA-1	20.0	50.0	20.0	50.0	20.0	50.0
Koumine	GA-2	1.0 (0.5)^*^	5.0 (1.0)	1.0 (0.5)	5.0 (1.0)	1.0 (0.5)	5.0 (1.0)
Gelsemine	GA-4	1.0 (0.5)	5.0 (1.0)	1.0 (0.5)	5.0 (1.0)	1.0 (0.5)	5.0 (1.0)
Na-desmethoxyhumantenine	GA-5	20.0	50.0	20.0	50.0	20.0	50.0
Gelsenicine	GA-6	1.0 (0.5)	5.0 (1.0)	1.0 (0.5)	5.0 (1.0)	1.0 (0.5)	5.0 (1.0)
Nb-Methylgelsedilam	GA-7	10.0	20.0	10.0	20.0	10.0	20.0
19-Hydroxdihyogelsemine	GA-9	5.0	10.0	5.0	10.0	5.0	10.0
14-Hydroxygelsenicine	GA-10	1.0	5.0	1.0	5.0	1.0	5.0
11-Hydroxygelsenicine	GA-11	10.0	20.0	10.0	20.0	10.0	20.0
Gelsevirine	GA-12	1.0	5.0	1.0	5.0	1.0	5.0
Isomer of Akuammidine	GA-14	1.0	5.0	1.0	5.0	1.0	5.0
Humantenine	GA-15	1.0	5.0	1.0	5.0	1.0	5.0
Gelsemicine	GA-16	1.0	5.0	1.0	5.0	1.0	5.0
Gelsemoxonine	GA-17	20.0	50.0	20.0	50.0	20.0	50.0
Humantenoxenine	GA-19	5.0	10.0	5.0	10.0	5.0	10.0
15-Hydroxyhumantenine	GA-20	1.0	5.0	1.0	5.0	1.0	5.0
19-Hydroxydigelsevirine	GA-21	10.0	20.0	10.0	20.0	10.0	20.0
6-Hydroxyhumantenine	GA-22	1.0	5.0	1.0	5.0	1.0	5.0
GS-2 (11-Methoxy-14-Hydroxygelsenicine)	GA-23	10.0	20.0	10.0	20.0	10.0	20.0
14-Dehydrxoygelsefuranidine	GA-24	10.0	20.0	10.0	20.0	10.0	20.0
Isomer of Dehydrxoygelsefuranidine (1)	GA-25	20.0	50.0	20.0	50.0	20.0	50.0
Isomer of Dehydrxoygelsefuranidine (2)	GA-26	20.0	50.0	20.0	50.0	20.0	50.0
11-Methoxy-19-hydroxygelsegine	GA-27	10.0	20.0	10.0	20.0	10.0	20.0

* , results of precise quantitative method.

Furthermore, the accuracy and precision results are shown in the form of a heat map in Supplementary Figure 5 and show that the accuracy of all analytes was within 80–120% and the deviation was < 20%. The matrix effects of all analytes in swine muscle, liver, and kidney tissues are shown in Supplementary Table 2. The results showed low matrix interference, and the RSD of all detected alkaloids was lower than 15%. The above results support that the semi-quantitation method is of reasonable accuracy and is applicable to the multicomponent quantitative analysis of *G. elegans* alkaloids.

After validation of the developed semiquantitative method, we extended the method for detection in other tissues and performed simple method verification, such as linear regression equations and LOQs in urine, bile, and other pig tissue samples; the results are shown in **Table 3**. Overall, the results were satisfactory and highlight that this semiquantitative method could be applied for the multicomponent quantification of *G. elegans* alkaloids in other pig tissues.

### 3.3. Tissue residue depletion of multiple components *G. elegans* alkaloids

To evaluate the residue depletion of multiple components *of G. elegans* alkaloids in various tissues of pigs during long-term feeding and after long-term drug administration, we utilized two efficient and validated UPLC-MS/MS approaches. Five types of alkaloids were detected in the liver, kidney, pancreas, brain, small intestine, and adrenal gland tissues; four types of alkaloids were detected in the muscle, heart, spleen, lungs, spinal cord, bile, and urine; and only three types of alkaloids were detected in the plasma.

### 3.4. Residue depletion in muscle, heart, and spleen

As presented in Table 2, 11 alkaloids belonging to the gelsemine-type (19-hydroxdihyogelsemine and 19-hydroxydigelsevirine), koumine-type (koumine), humantenine-type (15-hydroxyhumantenine), gelsedine-type (14-hydroxygelsenicine, 11-hydroxygelsenicine, gelsemoxonine, GS-2 (11-Methoxy-14-hydroxygelsenicine), and 11-methoxy-19-hydroxygelsegine) alkaloid groups were detected in pig muscle tissue. We found that 14-hydroxygelsenicine, 11-hydroxygelsenicine, gelsemoxonine, and GS-2 had the longest residence times in the muscle tissue (1 d), while the other seven alkaloids were not detected 1 d after drug withdrawal. From the muscle sample analytical results obtained using the established method, 11-hydroxygelsenicine was observed at the highest level (119.8 μg/kg) in the muscle samples, and 15-hydroxyhumantenine was observed at the lowest level (1.0 μg/kg) in the muscle samples.

**Table 2:** Concentrations of *Gelsemium* alkaloids in pig muscle, heart, and spleen after 45 d of consecutive administered *G. elegans* powder at a dose of 2% per kilogram feed (μg/kg) (*n* = 5).

**Compounds**	**Muscle (**μ**g/kg)**		**Heart (**μ**g/kg)**		**Spleen (**μ**g/kg)**	
	**6 h**	**1 d**	**6 h**	**1 d**	**6 h**	**1 d**
GA-1	ND	ND	ND	ND	ND	ND
GA-2 (^*^koumine)	1.5 ± 1.0 (1.60 ± 1.30)	ND (ND)	1.3 ± 1.4 (3.2 ± 4.0)	ND (2.0 ± 1.4)	3.5 ± 1.6 (3.7 ± 3.4)	ND (ND)
GA-4 (^*^gelsemine)	3.7 ± 2.7 (10.4 ± 7.6)	ND (ND)	7.1 ± 3.9 (30.6 ± 17.3)	ND (ND)	8.5 ± 5.6 (23.4 ± 15.4)	ND (1.8 ± 0.9)
GA-5	ND	ND	2.4 ± 1.3	ND	6.8 ± 3.8	ND
GA-6 (^*^gelsenicine)	2.1 ± 1.4 (ND)	ND (ND)	2.1 ± 1.8 (16.3 ± 13.6)	ND (ND)	3.9 ± 2.9 (1.4 ± 1.5)	ND (ND)
GA-7	ND	ND	1.0 ± 0.8	ND	1.7 ± 1.3	ND
GA-9	4.1 ± 1.7	ND	3.1 ± 1.6	ND	14.6 ± 6.9	ND
GA-10	4.4 ± 2.0	3.4 ± 2.0	83.6 ± 38.5	12.8 ± 8.6	89.8 ± 55.4	10.5 ± 7.6
GA-11	119.7 ± 66.4	19.6 ± 14.8	9.2 ± 4.6	1.8 ± 2.0	59.2 ± 25.0	4.3 ± 4.4
GA-12	ND	ND	1.0 ± 0.9	ND	ND	ND
GA-14	ND	ND	ND	ND	ND	ND
GA-15	ND	ND	ND	ND	2.4 ± 2.0	ND
GA-16	ND	ND	ND	ND	ND	ND
GA-17	60.4 ± 27.7	9.4 ± 4.2	56.0 ± 21.5	7.8 ± 3.7	54.3 ± 28.4	6.5 ± 2.9
GA-19	ND	ND	ND	ND	ND	ND
GA-20	1.0 ± 0.5	ND	ND	ND	4.6 ± 1.9	ND
GA-21	1.8 ± 1.5	ND	1.1 ± 0.8	ND	5.0 ± 3.9	ND
GA-22	ND	ND	ND	ND	ND	ND
GA-23	5.6 ± 0.9	1.2 ± 0.8	3.9 ± 1.3	ND	7.0 ± 2.9	1.5 ± 0.8
GA-24/25	ND	ND	ND	ND	ND	ND
GA-27	1.6 ± 0.8	ND	ND	ND	4.4 ± 3.2	ND

* , represents results of the precise quantitative method; ND, not detected.

A total of 12 alkaloids (gelsemine-type, koumine-type, humantenine-type, and gelsedine-type alkaloids) were detected in heart tissue samples. Among these alkaloids, the longest residence time in the heart tissue samples was that of 14-hydroxygelsenicine, 11-hydroxygelsenicine, and gelsemoxonine (1 d), whereas the other nine alkaloids had a residence time of 6 h. In addition, 14-hydroxygelsenicine and Nb-methylgelsedilam had the highest and lowest residual levels, respectively, in heart tissue samples 6 h after withdrawal.

Fourteen alkaloids were detected in the spleen tissue samples (gelsemine-type, koumine-type, humantenine-type, and gelsedine-type alkaloids) (Table 2). We observed these results and found that 14-hydroxygelsenicine, 11-hydroxygelsenicine, gelsemoxonine and GS-2 (belonging to gelsedine-type) had the longest residence time (1 d) in the spleen tissue samples, while the other ten alkaloids remained for only 6 h. 14-Hydroxygelsenicine and gelsenicine showed the highest (89.8 μg/kg) and lowest (1.4 μg/kg) residual amounts, respectively, in the lung tissue 6 h after withdrawal.

### 3.5. Residue depletion in the liver and kidney

The concentrations of alkaloids in liver tissue samples are listed in Table 3. These results revealed that 19 alkaloids were detected in the liver tissue. Sarpagine-type (koumidine), humantenine-type (15-hydroxyhumantenine), and gelsedine-type (14-hydroxygelsenicine and gelsemoxonine) alkaloids were still detectable in the liver tissue 3 d after withdrawal, whereas gelsemine-type and koumine-type alkaloids were not detected beyond 1 d after withdrawal. The residual amounts of sarpagine-type, humantenine-type, and gelsedine-type alkaloids decreased with increasing withdrawal time. In general, the residual levels of the 16 alkaloids were low in the liver tissue, with the exception of gelsemine, 19-hydroxdihyogelsemine, and 11-hydroxygelsenicine. The highest concentration was observed for 11-hydroxygelsenicine, followed by that of (in decreasing order) gelsemine, 19-hydroxdihyogelsemine, 6-hydroxyhumantenine, 19-hydroxydigelsevirine, Nb-methylgelsedilam and humantenine.

**Table 3:** Concentrations of *Gelsemium* alkaloids in pig liver and kidney after 45 d of consecutive administered *G. elegans* powder at a dose of 2% per kilogram feed (μg/kg) (*n* = 5).

**Compounds**	**Liver (**μ**g/kg)**			**Kidney (**μ**g/kg)**		
	**6 h**	**1 d**	**3 d**	**6 h**	**1 d**	**3 d**
GA-1	5.8 ± 2.1	2.3 ± 0.7	1.9 ± 0.7	4.7 ± 1.0	4.6 ± 3.1	ND
GA-2 (^*^koumine)	11.5 ± 5.6 (5.9 ± 4.9)	ND (1.1 ± 1.2)	ND (ND)	7.9 ± 4.1 (4.5 ± 3.1)	ND (2.0 ± 1.1)	ND (ND)
GA-4 (^*^gelsemine)	17.9 ± 11.3 (27.0 ± 17.3)	ND (ND)	ND (ND)	19.7 ± 11.6 (32.6 ± 21.8)	1.1 ± 1.1 (ND)	ND (ND)
GA-5	16.0 ± 5.8	2.5 ± 1.7	ND	20.0 ± 8.6	1.5 ± 1.7	ND
GA-6 (^*^gelsenicine)	10.5 ± 4.0 (3.6 ± 1.9)	ND (ND)	ND (ND)	7.0 ± 4.7 (2.3 ± 2.8)	ND (ND)	ND (ND)
GA-7	5.3 ± 2.0	ND	ND	3.4 ± 2.5	ND	ND
GA-9	25.3 ± 9.4	ND	ND	19.7 ± 8.1	1.1 ± 1.4	ND
GA-10	200.6 ± 108.7	25.2 ± 15.7	1.1 ± 1.3	196.5 ± 97.5	29.0 ± 17.5	1.0 ± 1.2
GA-11	409.0 ± 145.3	72.9 ± 50.1	ND	345.2 ± 131.5	92.2 ± 95.2	3.7 ± 2.6
GA-12	2.3 ± 1.2	ND	ND	1.2 ± 0.8	ND	ND
GA-14	ND	ND	ND	ND	ND	ND
GA-15	22.4 ± 7.9	4.0 ± 3.3	ND	5.0 ± 3.0	ND	ND
GA-16	1.8 ± 0.6	ND	ND	1.6 ± 0.6	ND	ND
GA-17	145.1 ± 67.2	19.3 ± 8.3	1.4 ± 1.6	183.3 ± 74.7	22.9 ± 11.4	ND
GA-19	2.0 ± 0.9	ND	ND	ND	ND	ND
GA-20	45.8 ± 26.7	12.3 ± 5.0	1.5 ± 1.1	11.6 ± 4.8	4.0 ± 4.4	ND
GA-21	7.9 ± 5.0	ND	ND	9.2 ± 0.2	ND	ND
GA-22	8.6 ± 3.8	ND	ND	2.1 ± 0.8	ND	ND
GA-23	7.5 ± 4.6	2.0 ± 1.4	ND	25.2 ± 8.8	5.3 ± 3.0	ND
GA-24/25	ND	ND	ND	ND	ND	ND
GA-27	5.0 ± 2.6	ND	ND	8.7 ± 4.1	ND	ND

* Represents results of the precise quantitative method; ND, not detected.

Eighteen alkaloids (including sarpagine-type, gelsemine-type, koumine-type, humantenine-type, and gelsedine-type alkaloids) were detected in kidney tissue samples. Notably, among the detected alkaloids, the longest residence time of 3 d was observed for gelsedine-type alkaloids (14-hydroxygelsenicine and 11-hydroxygelsenicine). Alkaloid residues in the kidney tissue samples gradually decreased over time. Moreover, we found that the most abundant alkaloid in the kidney samples was 11-hydroxygelsenicine (345.2 μg/kg) 6 h after withdrawal, and the lowest was 14-hydroxygelsenicine (1.0 μg/kg) 3 d after withdrawal.

### 3.6. Residue depletion in the lung

**Figure 1:**
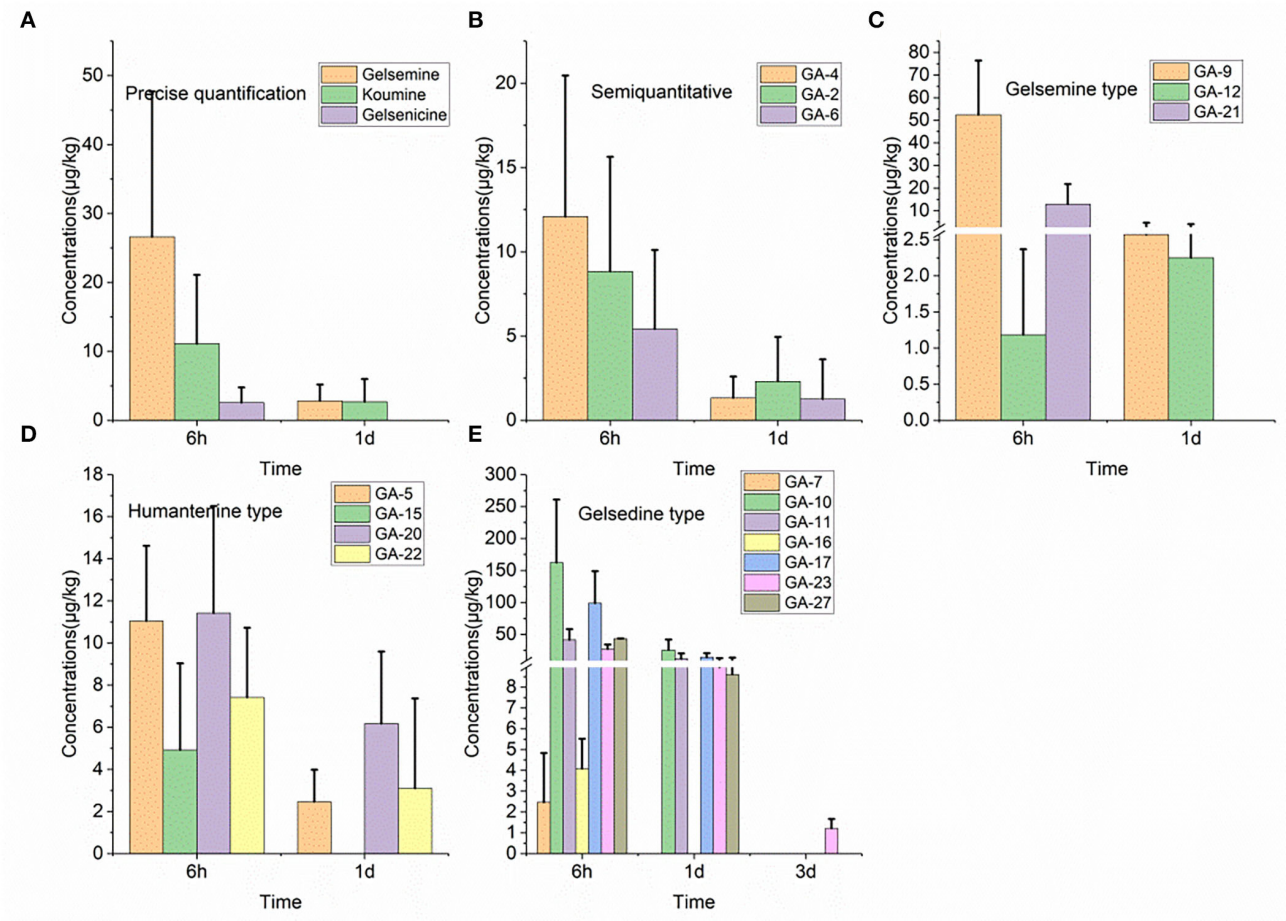
**(A–E)** Depletion of *Gelsemium* alkaloids in pig lung after 45 d of consecutive administered *G. elegans* powder at a dose of 2% per kilogram feed.

As shown in Figure 1, we detected 17 alkaloids (gelsemine-type, koumine-type, humantenine-type, and gelsedine-type alkaloids) in the lung tissue samples. From these results, we found that GS-2 remained in the lung tissue for the longest time (3 d), whereas five alkaloids, including gelsenicine, Nb-methylgelsedilam, humantenine, gelsemicine, and 19-hydroxydigelsevirine, remained in the lung tissue for only 6 h. 14-hydroxygelsenicine and gelsevirine exhibited the highest (162.5 μg/kg) and lowest (1.2 μg/kg) residual amounts, respectively, in the lung tissue samples 6 h after withdrawal.

### 3.7. Residue depletion in the pancreas

**Figure 2:**
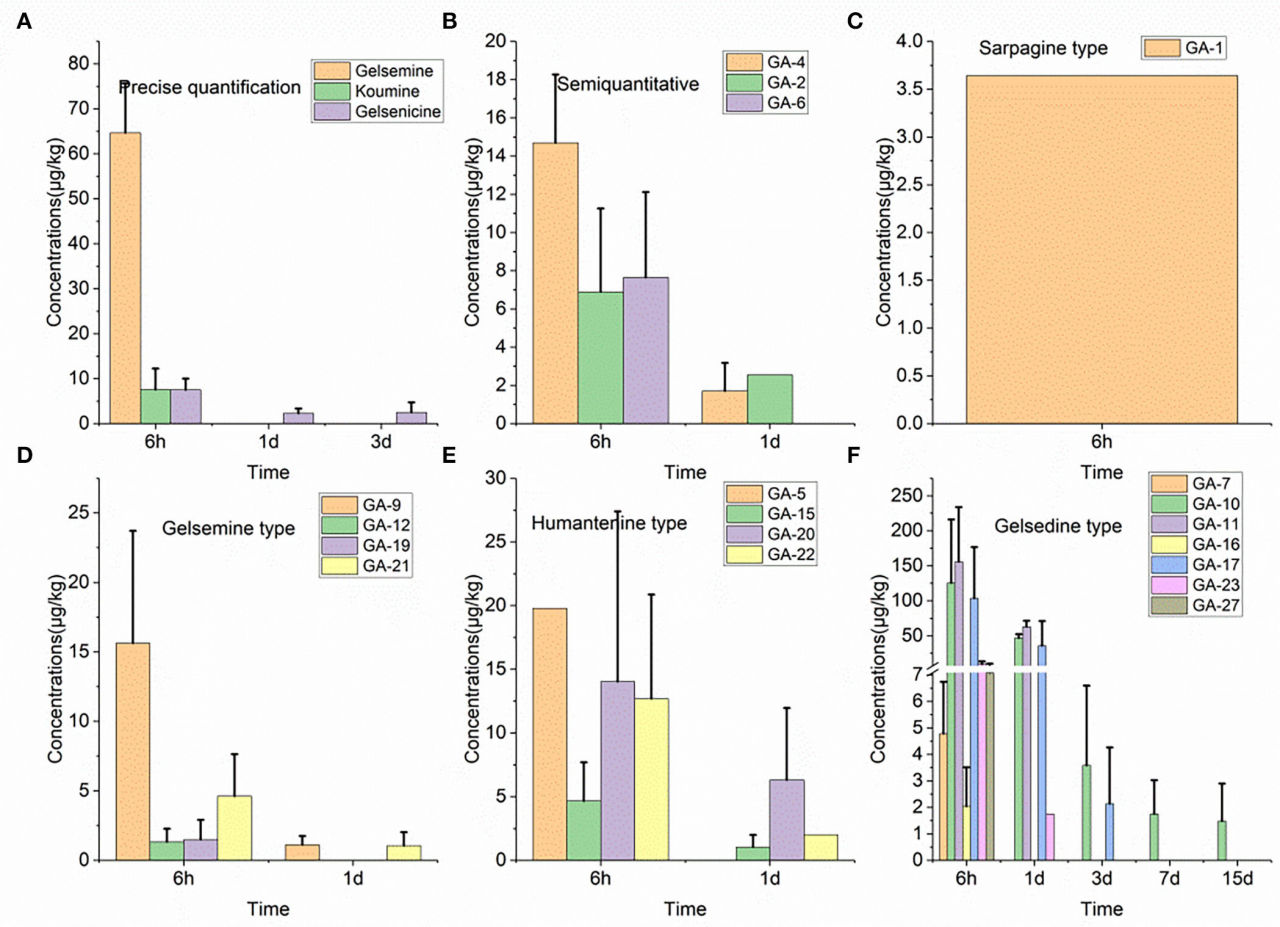
**(A–F)** Depletion of *Gelsemium* alkaloids in pig pancreas after 45 d of consecutive administered *G. elegans* powder at a dose of 2% per kilogram feed.

Nineteen alkaloid residue concentrations in pig pancreatic tissue samples are depicted in Figure 2. Based on these results, we found that 14-hydroxygelsenicine exhibited the longest residence time (15 d), while nine alkaloids, including koumidine, gelsemine, gelsevirine, humantenoxenine, koumine, Na-desmethoxyhumantenine, Nb-methylgelsedilam, gelsemicine, and 11-methoxy-19-hydroxygelsegine, had a residence time of only 6 h. The highest residual concentration of the nineteen alkaloids was that of 11-hydroxygelsenicine (155.7 μg/kg) 6 h after withdrawal, and humantenine exhibited the lowest residual amount (1.1 μg/kg) at 1 d after withdrawal. In addition, most of the alkaloids were eliminated 3 d after withdrawal.

### 3.8. Residue depletion in the brain and adrenal gland

We detected 14 alkaloids in pig brain tissue samples (Table 4). These results were comprehensively analyzed, and it was noted that low residual concentrations were detected in the brain tissue samples 3 d after withdrawal. However, most alkaloids were eliminated from the brain tissue after 6 h. Results showed that the concentrations of 14-hydroxygelsenicine and 6-hydroxyhumantenine at 6 h were the highest (48.4 μg/kg) and lowest (1.5 μg/kg) in the brain samples, respectively.

**Table 4:** Concentrations of *Gelsemium* alkaloids in pig brain and adrenal gland after 45 d of consecutive administered *G. elegans* powder at a dose of 2% per kilogram feed (μg/kg) (*n* = 5).

**Compounds**	**Brain (**μ**g/kg)**			**Adrenal gland (**μ**g/kg)**		
	**6 h**	**1 d**	**3 d**	**6 h**	**1 d**	**3 d**
GA-1	1.6 ± 0.6	2.3 ± 3.1	ND	1.5 ± 0.9	3.5 ± 1.9	2.4 ± 2.8
GA-2 (^*^koumine)	3.6 ± 2.1 (3.2 ± 2.3)	ND (ND)	ND (ND)	3.0 ± 2.4 (2.9 ± 2.6)	ND (ND)	ND (ND)
GA-4 (^*^gelsemine)	10.0 ± 4.4 (23.8 ± 10.9)	3.9 ± 2.1 (10.2 ± 2.4)	1.4 ± 1.1 (2.4 ± 2.7)	7.6 ± 5.1 (227.0 ± 159.3)	ND (3.9 ± 0.8)	ND (ND)
GA-5	2.8 ± 1.5	ND	ND	5.5 ± 5.0	ND	ND
GA-6 (^*^gelsenicine)	3.3 ± 2.1 (1.6 ± 1.2)	ND (ND)	ND (ND)	2.7 ± 1.4 (1.4 ± 0.8)	ND (ND)	ND (ND)
GA-7	1.7 ± 1.1	ND	ND	2.6 ± 1.3	ND	ND
GA-9	8.1 ± 3.5	ND	ND	11.3 ± 4.4	ND	ND
GA-10	48.4 ± 28.7	5.9 ± 3.7	ND	51.3 ± 27.3	21.3 ± 13.9	2.5 ± 3.8
GA-11	4.8 ± 0.7	ND	ND	218.2 ± 115.0	52.4 ± 45.5	ND
GA-12	ND	ND	ND	ND	ND	ND
GA-14	ND	ND	ND	ND	ND	ND
GA-15	2.8 ± 1.6	ND	ND	2.3 ± 1.4	ND	ND
GA-16	ND	ND	ND	ND	ND	ND
GA-17	27.6 ± 13.1	3.1 ± 1.2	ND	35.9 ± 16.4	16.57 ± 9.5	2.5 ± 3.2
GA-19	ND	ND	ND	ND	ND	ND
GA-20	1.1 ± 0.3	ND	ND	3.8 ± 2.3	2.4 ± 1.9	ND
GA-21	ND	ND	ND	2.6 ± 1.8	ND	ND
GA-22	1.5 ± 0.5	ND	ND	2.7 ± 2.3	ND	ND
GA-23	3.3 ± 0.3	ND	ND	5.2 ± 1.7	3.0 ± 1.6	ND
GA-24/25	ND	ND	ND	ND	ND	ND
GA-27	ND	ND	ND	4.4 ± 2.5	ND	ND

* , represents results of the precise quantitative method; ND, not detected.

As described in Table 4, 16 alkaloids were detected in pig adrenal gland tissue samples. These results revealed that koumidine, 14-hydroxygelsenicine, and gelsemoxonine were still present in the adrenal gland tissue 3 d after withdrawal, while the other alkaloids were completely eliminated or below the LOQs 1 day after withdrawal. Moreover, 11-hydroxygelsenicine detected in the adrenal gland tissue presented the largest residual amount 6 h after withdrawal, whereas gelsenicine exhibited the lowest residual amount at the same time point.

### 3.9. Residue depletion in the small intestine

**Figure 3:**
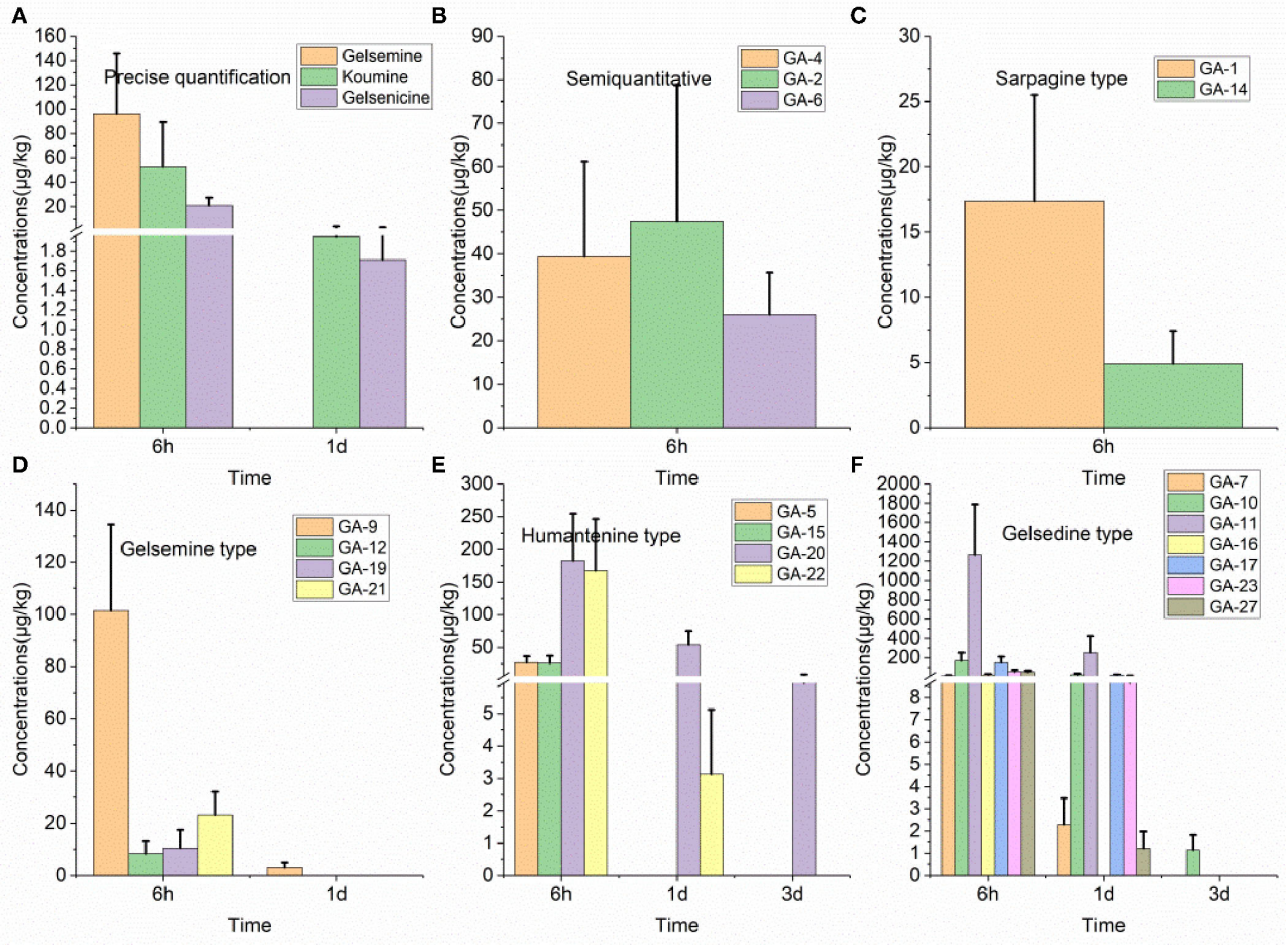
**(A–F)** Depletion of *Gelsemium* alkaloids in pig small intestine after 45 d of consecutive administered *G. elegans* powder at a dose of 2% per kilogram feed.

Twenty alkaloids were detected in the small intestine tissue samples of swine, which was the highest number of detected alkaloids in any of the analyzed tissues, as presented in Figure 3. Further analysis of these results revealed that 14-hydroxygelsenicine and 15-hydroxyhumantenine remained in the small intestine of pigs for 3 d, while most other alkaloids were not detected after 1 d. In this depletion study, 6 h after withdrawal, the 11-hydroxygelsenicine and 14-hydroxygelsenicine residue levels were highest (1,265.4 μg/kg) and the lowest (1.2 μg/kg), respectively, in the small intestine 3 d after withdrawal.

### 3.10. Residue depletion in the spinal cord

As presented in Table 5, we detected 14 alkaloids in spinal cord tissue samples. These results indicated that 14-hydroxygelsenicine, a gelsedine-type alkaloid, could remain in the spinal cord for up to 3 d after withdrawal, whereas the other 13 alkaloids were eliminated after 1 d. The residual concentration level of 14-hydroxygelsenicine was the highest (98.3 μg/kg) 6 h after withdrawal, while 11-methoxy-19-hydroxygelsegine presented the lowest concentration (1.0 μg/kg) at the same time point.

**Table 5:** Concentrations of *Gelsemium* alkaloids in pig spinal cord and bile after 45 d of consecutive administered *G. elegans* powder at a dose of 2% per kilogram feed (μg/kg) (*n* = 5).

**Compounds**	**Spinalcord (**μ**g/kg)**			**Bile (**μ**g/kg)**			
	**6h**	**1d**	**3d**	**6h**	**1d**	**3d**	**7d**
GA-1	5.3 ± 3.1 (5.2 ± 2.7)	1.4 ± 0.3 (ND)	1.1 ± 0 (ND)	ND	ND	ND	ND
GA-2 (^*^koumine)	11.6 ± 7.6 (17.2 ± 14.1)	7.3 ± 1.7 (ND)	ND (ND)	ND (22.9 ± 13.0)	ND (1.6 ± 1.7)	ND (ND)	ND (ND)
GA-4 (^*^gelsemine)	ND	ND	ND	ND	ND	ND	ND
GA-5	4.2 ± 2.0 (3.2 ± 1.5)	ND (ND)	ND (ND)	2.9 ± 0.0 (110.1 ± 80.6)	8.5 ± 4.8 (4.4 ± 3.0)	ND (ND)	ND (ND)
GA-6 (^*^gelsenicine)	ND	ND	ND	1.3 ± 0.0	ND	ND	ND
GA-7	6.5 ± 3.7	ND	ND	ND (15.0 ± 4.3)	ND (1.9 ± 1.2)	ND (ND)	ND (ND)
GA-9	98.3 ± 71.6	25.9 ± 19.1	1.1 ± 0.9	3.0 ± 4.2	3.9 ± 2.6	2.6 ± 1.3	ND
GA-10	18.0 ± 9.9	ND	ND	ND	ND	ND	ND
GA-11	1.3 ± 0.7	ND	ND	6.5 ± 11.1	13.0 ± 10.8	2.4 ± 1.1	ND
GA-12	ND	ND	ND	83.3 ± 0.0	121.4 ± 89.5	15.8 ± 17.0	16.0 ± 0.0
GA-14	2.8 ± 1.6	1.1 ± 0.4	ND	ND	ND	ND	ND
GA-15	ND	ND	ND	3.4 ± 0.0	7.5 ± 2.9	ND	ND
GA-16	84.8 ± 57.2	17.4 ± 14.5	ND	ND	ND	ND	ND
GA-17	ND	ND	ND	ND	ND	ND	ND
GA-19	2.4 ± 2.2	1.0 ± 0.3	ND	ND	ND	ND	ND
GA-20	1.4 ± 0.9	ND	ND	67.7 ± 0.0	85.7 ± 65.1	7.7 ± 7.9	5.6 ± 0.0
GA-21	1.4 ± 1.2	ND	ND	1.4 ± 0.0	1.0 ± 0.5	ND	ND
GA-22	5.3 ± 2.8	2.4 ± 0.8	ND	1.7 ± 2.3	3.2 ± 21	ND	ND
GA-23	ND	ND	ND	7.3 ± 0.0	12.2 ± 10.4	ND	ND
GA-24/25	1.0 ± 0.9	ND	ND	ND	ND	ND	ND
GA-27	5.3 ± 3.1 (5.2 ± 2.7)	1.4 ± 0.3 (ND)	1.1 ± 0 (ND)	4.9 ± 8.4	10.9 ± 5.2	6.2 ± 5.6	4.7 ± 3.9

* , represents results of the precise quantitative method; ND, not detected.

### 3.11. Residue depletion in bile

As shown in Table 5, 13 alkaloids were detected in bile samples. Among them, 11-hydroxygelsenicine, gelsemoxonine, and GS-2, which are gelsedine-type alkaloids, showed the longest residence time in the bile (7 d), whereas humantenine-type Na-desmethoxyhumantenine presented the shortest residence time (6 h). Other alkaloids were eliminated after 1 and 3 d of withdrawal. These results revealed that 11-hydroxygelsenicine and humantenoxenine showed the highest (121.4 μg/kg) and lowest (1.0 μg/kg) residues, respectively, in bile 1 d after withdrawal.

### 3.12. Depletion in urine

**Figure 4:**
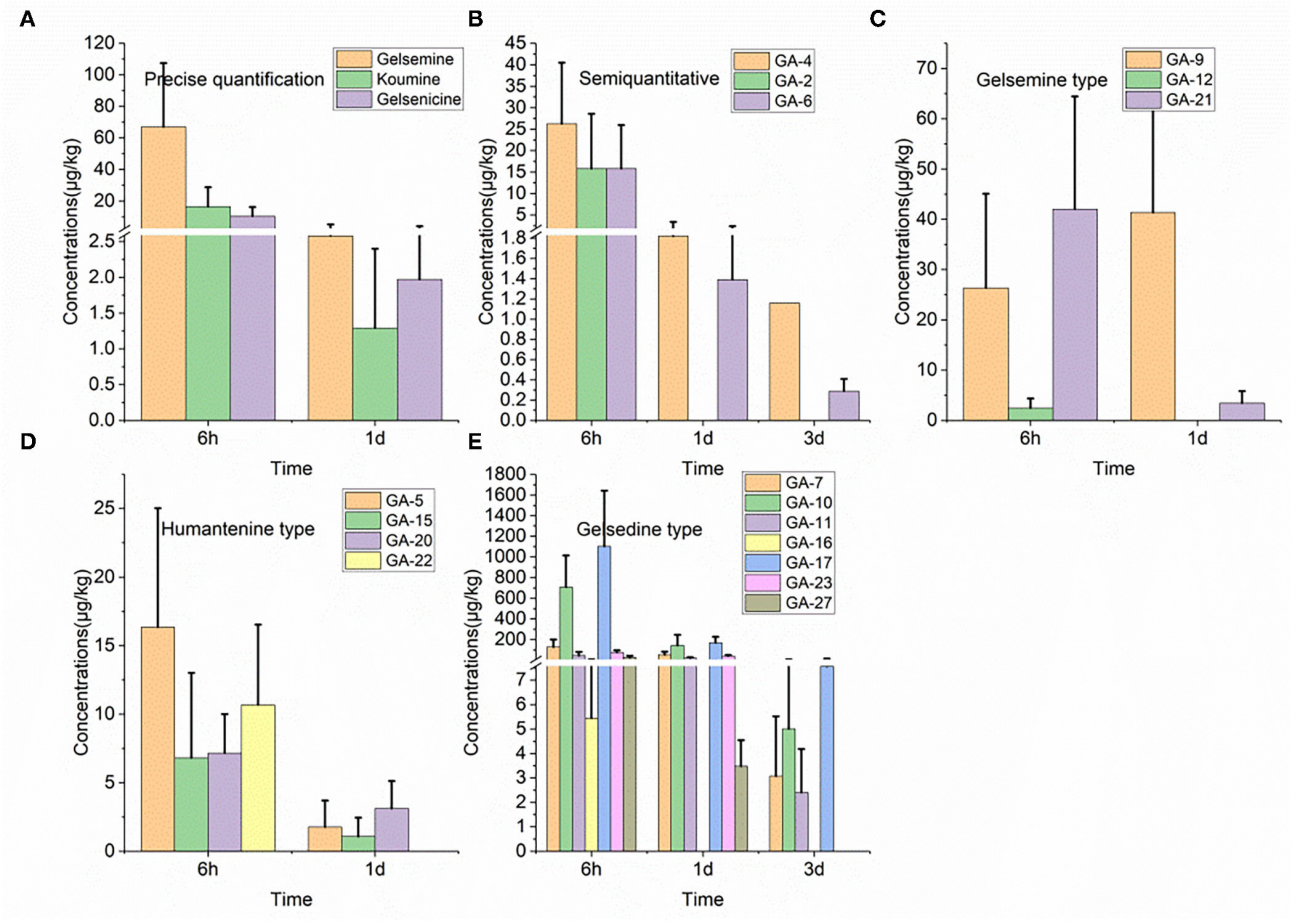
**(A–E)** Depletion of *Gelsemium* alkaloids in pig urine after 45 d of consecutive administered *G. elegans* powder at a dose of 2% per kilogram feed.

Seventeen alkaloids were detected in urine tissue samples, as shown in Figure 4. In this study, the gelsedine-type alkaloid residues, Nb-methylgelsedilam, 14-hydroxygelsenicine, 11-hydroxygelsenicine, and gelsemoxonine, in urine samples were eliminated very slowly, and their estimated withdrawal time was 3 d, which was longer than that of the other alkaloids described above. Six hours after withdrawal, the gelsemoxonine residue level was highest (1,102.6 μg/kg) in the urine tissue, whereas 1 d after withdrawal, the humantenine residual concentration was lowest (1.1 μg/kg) in urine.

### 3.13. Depletion in plasma

**Figure 5:**
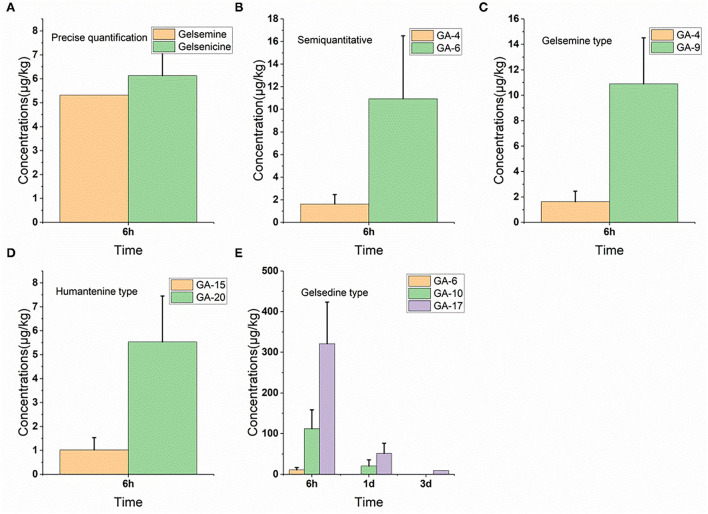
**(A–E)** Depletion of *Gelsemium* alkaloids in pig plasma after 45 d of consecutive administered *G. elegans* powder at a dose of 2% per kilogram feed.

As depicted in Figure 5, only seven alkaloids (gelsemine-type, humantenine-type, and gelsedine-type alkaloids) were detected in the plasma samples. However, in the plasma, only gelsemoxonine of gelsedine-type residues was eliminated slowly, as they were detected in the plasma 3 d after withdrawal. The other five alkaloids (gelsemine, gelsenicine, 19-hydroxdihyogelsemine, humantenine, and 15-hydroxyhumantenine) remained in the plasma for only 6 h. We observed that 6 h after withdrawal, gelsemoxonine and humantenine residue levels were highest (321.1 μg/kg) and lowest (1.0 μg/kg) in the plasma samples, respectively.

### 3.14. Precise quantitative tissue residue depletion of gelsemine, koumine and gelsenicine

To confirm that the new semi-quantification assay provided accurate residue measurements of the components, the results obtained from the three *G. elegans* alkaloids with authentic standards in various tissues (muscle, liver, kidneys, heart, spleen, lungs, pancreas, brain, small intestine, adrenal gland, spinal cord, bile, urine, plasma, and gonads) were compared with those from the semi-quantification assays. The results indicated that the quantitative performance of the two approaches remained consistent in all tissue matrices other than those of the spinal cord and bile. According to these results, the optimized semi-quantification method described herein was reliable to some extent and suitable for determining multiple *G. elegans* alkaloid residues in tissues independent of standards.

### 3.15. Correlation of 14-hydroxygelsenicine and gelsemoxonine concentration between tissues and biological fluids tissues in pigs

In pigs, the correlations of 14-hydroxygelsenicine and gelsemoxonine concentrations in edible tissues with those in urine and plasma were analyzed using linear regression. Excellent correlations were observed when linear functional equations were used to describe the relationships between the 14-hydroxygelsenicine and gelsemoxonine concentrations in the assayed edible tissues, urine, and plasma in pigs. The correlation coefficients of 14-hydroxygelsenicine ranged from 0.96 to 0.99 between the concentration in the liver, kidneys, small intestine, and pancreas and that in the urine (Supplementary Figures 6A–D), whereas the correlation coefficients of gelsemoxonine ranged from 0.93 to 0.99 between the concentration in the liver and pancreas, and that in the urine (Supplementary Figures 6E, F). In addition, the correlation coefficients ranged from 0.92 to 0.99 for gelsemoxonine between the concentration in the liver and pancreas, and that in the plasma (Supplementary Figures 6G, H). No correlations were observed for the muscle, brain, and lungs, because 14-hydroxygelsenicine and gelsemoxonine were only detected at the two earliest sampling times.

## 4. Discussion

PFAs have become a trend in animal husbandry feed after the ban of the use of antibiotics as growth promoters (2, 31, 32). However, researchers have mainly focused on their clinical pharmacological effects, such as antioxidant (6) and antibacterial activities (8), and their intestinal activity promotion (11), and believe that plant extracts are safe to add and have no adverse effects. Since the complexity of plant extracts and the difficulty in accurate quantification without standard products, little attention has been paid to their residues in animal-derived foods. Our previous study showed that after a single oral dose of *G. elegans* to pigs (1.0 g/kg) and rats (0.1 g/kg), alkaloids of it were rapidly absorbed in pigs and rats. The elimination rates of these alkaloids in pigs were slower than those in rats (17). Owing to the lack of a variety of *G. elegans* alkaloid standards, it is difficult to study the tissue distribution characteristics of *G. elegans*. At present, research on *G. elegans* mainly focuses on the pharmacokinetics and tissue distribution of the three alkaloid compounds in rats (25–27). However, only a few alkaloids contained in *G. elegans* do not fully reflect the overall characteristics of the plant. In this study, methods for multicomponent alkaloid LC-MS/MS detection in tissues were established and validated for the first time, and tissue depletion studies were successfully performed using these two methods.

Koumine had a higher content than the other alkaloids in the raw materials, but its toxicity was less than that of other alkaloids (LD_50_ in mice was 99 mg/kg) (14). Thus, many recent studies have focused on its pharmacological activity, such as anxiolytic (33), anxiolytic (34), and anti-inflammatory (35). But few studies have focused on its tissue distribution. However, in the present study, koumine was detected in tissues other than plasma. This alkaloid remained in the liver, kidneys, heart, lungs, small intestine, bile, and urine for 1 d and was eliminated after only 6 h in the other six tissues. Moreover, residual levels of koumine in the tissues gradually decreased over time in the small intestine, bile, urine, lungs, pancreas, liver, spinal cord, kidneys, spleen, brain, heart, adrenal gland, and muscle (52.64–1.60 μg/kg). These results indicate that the depletion rate of koumine in small intestine tissue is slow.

Gelsemine (a gelsemine-type alkaloid) is also more abundant than the other alkaloids in *G. elegans*, and few studies have been conducted on its residual depletion. After 3 d of withdrawal, gelsemine was only detected in the brain tissue, while it was not detected in the other tissues. Moreover, gelsemine showed the highest concentration (227.0 μg/kg) in paranephric tissues and the lowest concentration (5.32 μg/kg) in plasma. In addition, other gelsemine-type alkaloids, such as 19-hydroxdihyogelsemine, gelsevirine, humantenoxenine, and 19-hydroxydigelsevirine, exhibited residence times of < 3 d in all tissues. Among them, 19-hydroxdihyogelsemine exhibited the lowest residue levels in most tissues, except in the small intestine, lungs, and urine. The highest concentration of this alkaloid was observed in the small intestine, followed by (in decreasing order) the lungs, urine, liver, kidneys, pancreas, spleen, adrenal gland, plasma, brain, spinal cord, muscle, and heart, while 19-hydroxdihyogelsemine was not detected in bile. Furthermore, the concentration of gelsevirine in the small intestine reached a maximum, while it reached a minimum at 6 h after withdrawal. Gelsevirine was not detected in the muscle, spleen, brain, adrenal gland, bile, or urine. Humantenoxenine was not detected in most tissues, except in the liver, pancreas, small intestine, and bile. Additionally, the 19-hydroxydigelsevirine residue concentration was low in most tissues, except in the urine and small intestine. The above results indicate that gelsemine-type alkaloids can be eliminated quickly in most tissues within 1 d of ceasing drug administration.

It has been previously reported that gelsedine-type alkaloids are the most toxic components in *G. elegans* based on the LD_50_ values of the compounds (14). For example, the LD_50_ values of gelsenicine and gelsemicine in mice and rats were 0.1–0.2 and 0.1–0.3 mg/kg, respectively. Eight gelsedine-type alkaloids were detected in the liver, kidneys, lungs, pancreas, small intestine, and urine, whereas seven gelsedine-type alkaloids were detected in the heart, brain, and spinal cord tissues. In addition, six gelsedine-type alkaloids were detected in the heart, brain, and spinal cord tissues, and five alkaloids were detected in muscle tissue. However, only three gelsedine-type alkaloids were detected in the plasma. To the best of our knowledge, gelsedine-type alkaloid residues have not yet been reported. The tissue residue results show that gelsedine-type alkaloids are widely distributed in various pig tissues. Most gelsedine-type alkaloids were detected in most tissues and excreta until 1 d after withdrawal, and the longest persistence was observed in the pancreas as 14-hydroxygelsenicine was still detectable 15 d after withdrawal. 14-hydroxygelsenicine exhibited the highest residual concentration in heart, spleen, lung, brain, and spinal cord tissues at all sampling times, among which the highest concentration was in the lung tissues (162.45 μg/kg) 6 h after withdrawal. Relatively higher residual concentrations of 11-hydroxygelsenicine were observed in the muscle, liver, kidneys, pancreas, small intestine, and bile at all sampling times. Notably, the 11-hydroxygelsenicine concentration in the small intestine (1,265.44 μg/kg) was much higher than that in the bile (121.36 μg/kg), pancreas (155.68 μg/kg) and muscle (119.75 μg/kg). Gelsemoxonine exhibited the highest residue concentrations in the urine and plasma of all gelsedine-type alkaloids. Based on the reported toxicity of this type of alkaloid, the consumption of animal-derived products from *G. elegans* feed poses risks to human health.

Plasma and urine can be used to monitor drug residues in edible tissues (36, 37). Plasma is considered the most appropriate biological fluid for *in vivo* drug monitoring because the drug concentration in the plasma represents an instantaneous concentration, which makes the estimated tissue concentration more meaningful for routine residue monitoring. Good correlations of gelsemoxonine concentrations between plasma, liver, and pancreatic tissues of pigs were observed, thereby indicating that plasma could be used to monitor the drug residues of gelsemoxonine in the edible tissues of pigs. Excellent correlations of 14-hydroxygelsenicine and gelsemoxonine concentrations between urine and other edible tissues of pigs were observed, which demonstrated that urine was a satisfactory method for monitoring 14-hydroxygelsenicine and gelsemoxonine residues.

## 5. Conclusion

In conclusion, we report for the first time the development and validation of a highly sensitive and specific method for the determination of multicomponent alkaloids independently of the standards. This validated detection method was successfully applied to tissue residue depletion studies and it was found that many *G. elegans* alkaloids were widely distributed in most pig tissues. In addition, 14-hydroxygelsenicine, 11-hydroxygelsenicine, and gelsemoxonine were selected for residue monitoring of *G. elegans*, and pancreas, small intestine, and lung tissues were observed as the potential target tissues of *G. elegans*. In addition, both urine and plasma can be used to monitor the application of *G. elegans* and predict tissue concentrations using the developed semi-quantification method since sample collection is convenient and harmless to animals. The present results provide scientific evidence for evaluating the safety of animal-derived food for consumers and will be helpful for its application and future development.

## Data availability statement

The original contributions presented in the study are included in the article/Supplementary material, further inquiries can be directed to the corresponding authors.

## Ethics statement

The animal study was reviewed and approved by Animal Care and Use Committee, Hunan Agricultural University.

## Author contributions

Z-LS and Z-YL conceived the idea and experiment. Z-YL and YW designed and performed the experiments and wrote the paper. Z-YL, YW, X-ML, G-FL, and XB analyzed the data. All authors contributed to the article and approved the submitted version.
