# Supplementary material for: The multicomponent residue depletion of Gelsemium elegans in pig tissues, urine, and plasma
Source: Front Vet Sci. 2023 Jan 13;9:1111782. doi: 10.3389/fvets.2022.1111782 (PMC9880259; doi:10.3389/fvets.2022.1111782)
Supplement: Supplementary file 1 [file Data_Sheet_1.docx]

**SUPPLEMENTAL MATERIAL**

The multicomponent residue depletion of *Gelsemium elegans* in pig tissues, urine and plasma

Yong Wu ^a,1#^, Kun Yang^a,1#^, Jun-Jie Cao^a^, Xue-Ming Long^b^, Gao-Feng Liu^c^, Xia Bai ^a^,

Zhi-Liang Sun^a *^, Zhao-Ying Liu ^a*^

^a^College of Veterinary Medicine, Hunan Agricultural University, 1 Nongda Rd, District Furong, Changsha city 410128, Hunan China.

^b^Hunan Provincial Institute of Veterinary Drugs and Feed Control, 61 Xiaoxiangzhong Rd, District Yuelu, Changsha city 410006, Hunan China.

^c^Hunan Canzoho Biological Technology Co. Ltd., 321 Kangning Rd, District Economic and technological development, Liuyang city 410329, Hunan China.

**^*^***Correspondence: sunzhiliang1965@aliyun.com(Z.L.Sun);*

*liu_zhaoying@hunau.edu.cn (Z.Y. Liu),Tel.: +86 731 8468189; fax: +86 731 8468189.*

**^1 #^ The authors contributed equally to this work.**


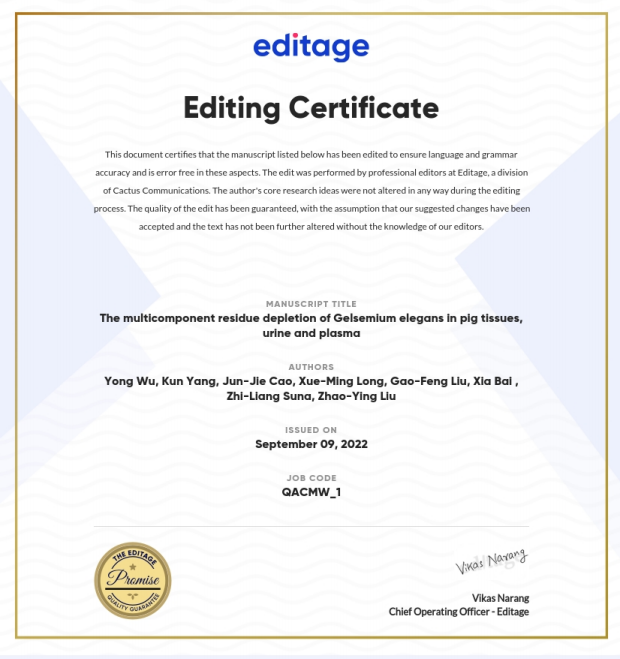


**TABLES**

**Table S1.** Result of calibration curve in pig tissues (muscle, liver and kidney) of three alkaloids and *Gelsemium* total alkaloids.

| **Tissues** | **Analytes** | **Linear range(μg/L)** | **Linearity** | **（R^2^）** |
| --- | --- | --- | --- | --- |
| Muscle | GA-1 | 50.0-200.0 | y=576x-2.4e+003 | 0.9950 |
|  | GA-2(*koumine) | 5.0-200.0(1.0-100.0) | y=1.15e+003x+148(*y=1.89e+003x+7.37e+003) | 0.9923(*0.9957) |
|  | GA-4(*gelsemine) | 5.0-200.0(1.0-100.0) | y=8.63e+003x+1.74 e+003(*y=8.77e+003x+8.89 e+003) | 0.9967(*0.9989) |
|  | GA-5 | 50.0-200.0 | y=222x-1.58e +003 | 0.9990 |
|  | GA-6(*gelsenicine) | 5.0-200.0(1.0-100.0) | y=1.07e+004x+1.28 e+003(*y=2.63e+004x+9.01 e+004 | 0.9987(*0.9951) |
|  | GA-7 | 20.0-200.0 | y=221x-288 | 0.9996 |
|  | GA-9 | 10.0-200.0 | y=608x-147 | 0.9953 |
|  | GA-10 | 5.0-200.0 | y=1.1e+003x+247 | 0.9980 |
|  | GA-11 | 20.0-200.0 | y=236x+169 | 0.9988 |
|  | GA-12 | 5.0-200.0 | y=1.06e+004x+433 | 0.9972 |
|  | GA-14 | 5.0-200.0 | y=1.15e+003x+2.03e+003 | 0.9916 |
|  | GA-15 | 5.0-200.0 | y=8.6e+003x-122 | 0.9998 |
|  | GA-16 | 5.0-200.0 | y=2.42e+003x-157 | 0.9998 |
|  | GA-17 | 50.0-200.0 | y=63.4x-271 | 0.9987 |
|  | GA-19 | 10.0-200.0 | y=743x-182 | 0.9973 |
|  | GA-20 | 5.0-200.0 | y=1.74e+003x+104 | 0.9998 |
|  | GA-21 | 20.0-200.0 | y=221x-266 | 0.9912 |
|  | GA-22 | 5.0-200.0 | y=621x+36.3 | 0.9985 |
|  | GA-23 | 20.0-200.0 | y=134x+211 | 0.9928 |
|  | GA-24 | 20.0-200.0 | y=382x-183 | 0.9944 |
|  | GA-25 | 50.0-200.0 | y=111x-1.41e +003 | 0.9928 |
|  | GA-26 | 50.0-200.0 | y=207x-2.29e +003 | 0.9905 |
|  | GA-27 | 20.0-200.0 | y=279x-243 | 0.9960 |
| Liver | GA-1 | 50.0-200.0 | y=453x-1.27e +003 | 0.9945 |
|  | GA-2(*koumine) | 5.0-200.0(1.0-100.0) | y=982x+254(y=1.53e+003x+4.79e+003) | 0.9987(0.9935) |
|  | GA-4(*gelsemine) | 5.0-200.0(1.0-100.0) | y=6.04e+003x+1.18 e+003(y=6.88e+003x+1.57 e+004) | 0.9979(0.9967) |
|  | GA-5 | 50.0-200.0 | y=165x+403 | 0.9980 |
|  | GA-6(*gelsenicine) | 5.0-200.0(1.0-100.0) | y=1.02e+004x+951(y=2.47e+004x+5.28e+004) | 0.9998(0.9982) |
|  | GA-7 | 20.0-200.0 | y=184x-73.5 | 0.9980 |
|  | GA-9 | 10.0-200.0 | y=583x-47 | 0.9988 |
|  | GA-10 | 5.0-200.0 | y=914x+44.4 | 0.9993 |
|  | GA-11 | 20.0-200.0 | y=202x-187 | 0.9983 |
|  | GA-12 | 5.0-200.0 | y=1.01e+004x+1.67e+003 | 0.9998 |
|  | GA-14 | 5.0-200.0 | y=904x-227 | 0.9948 |
|  | GA-15 | 5.0-200.0 | y=7.64e+003x+620 | 0.9997 |
|  | GA-16 | 5.0-200.0 | y=2.32e+003x+1.18e+003 | 0.9977 |
|  | GA-17 | 50.0-200.0 | y=52.1x-67.1 | 0.9920 |
|  | GA-19 | 10.0-200.0 | y=686x+912 | 0.9943 |
|  | GA-20 | 5.0-200.0 | y=1.5e+003x-72.2 | 0.9986 |
|  | GA-21 | 20.0-200.0 | y=193x+189 | 0.9960 |
|  | GA-22 | 5.0-200.0 | y=511x+51.1 | 0.9980 |
|  | GA-23 | 20.0-200.0 | y=114x-222 | 0.9982 |
|  | GA-24 | 20.0-200.0 | y=335x+11.6 | 0.9971 |
|  | GA-25 | 50.0-200.0 | y=96.9x+587 | 0.9982 |
|  | GA-26 | 50.0-200.0 | y=187x-294 | 0.9993 |
|  | GA-27 | 20.0-200.0 | y=252x-283 | 0.9992 |
| Kidney | GA-1 | 50.0-200.0 | y=442x+5.66e +003 | 0.9990 |
|  | GA-2(*koumine) | 5.0-200.0(1.0-100.0) | y=622x+363(2.39e+003x+6.81e+003) | 0.9997(0.9960) |
|  | GA-4(*gelsemine) | 5.0-200.0(1.0-100.0) | y=5.59e+003x+1.54e+003(8.33e+003x+2.13e+004) | 0.9979(0.9969) |
|  | GA-5 | 50.0-200.0 | y=152x+634 | 0.9928 |
|  | GA-6(*gelsenicine) | 5.0-200.0(1.0-100.0) | y=7.85e+003x+2.76e+003(3.04e+004x+1.04e+005) | 0.9985(0.9959) |
|  | GA-7 | 20.0-200.0 | y=165x-153 | 0.9981 |
|  | GA-9 | 10.0-200.0 | y=561x+299 | 0.9962 |
|  | GA-10 | 5.0-200.0 | y=1.02e+003x+123 | 0.9980 |
|  | GA-11 | 20.0-200.0 | y=282x+7.33 | 0.9973 |
|  | GA-12 | 5.0-200.0 | y=7.04e+003x+598 | 0.9998 |
|  | GA-14 | 5.0-200.0 | y=612x+176 | 0.9997 |
|  | GA-15 | 5.0-200.0 | y=7.1e+003x+2.46e+003 | 0.9992 |
|  | GA-16 | 5.0-200.0 | y=2.19e+003x+542 | 0.9981 |
|  | GA-17 | 50.0-200.0 | y=56.4x-163 | 0.9992 |
|  | GA-19 | 10.0-200.0 | y=665x+811 | 0.9990 |
|  | GA-20 | 5.0-200.0 | y=1.48e+003x+618 | 0.9996 |
|  | GA-21 | 20.0-200.0 | y=126x+567 | 0.9916 |
|  | GA-22 | 5.0-200.0 | y=547x+366 | 0.9991 |
|  | GA-23 | 20.0-200.0 | y=66.8x+260 | 0.9959 |
|  | GA-24 | 20.0-200.0 | y=313x-863 | 0.9951 |
|  | GA-25 | 50.0-200.0 | y=96.9x+587 | 0.9982 |
|  | GA-26 | 50.0-200.0 | y=187x-294 | 0.9993 |
|  | GA-27 | 20.0-200.0 | y=219x-388 | 0.9961 |

*: Represents the result of the precise quantitative method.

**Table S2.** Results of the matrix effects in pig muscle, liver and kidney tissues.

| **Analytes** | **Name** | **Muscle** | **Liver** | **Kidney** |
| --- | --- | --- | --- | --- |
| GA-1 | Koumidine | 18.05±6.42 | 9.06±4.67 | 8.49±2.80 |
| GA-2 | Koumine | 19.29±12.29(*10.90±3.50) | 13.98±3.79(13.70±4.00) | 9.57±5.49(13.50±4.40) |
| GA-4 | Gelesmine | 14.02±4.99(12.20±5.30) | 9.73±4.06(14.60±4.90) | 14.49±6.03(14.20±5.10) |
| GA-5 | Na-desmethoxyhumantenine | 6.08±6.07 | 7.68±3.73 | 7.44±4.25 |
| GA-6 | Gelsenicine | 13.62±5.85(11.80±2.70) | 10.07±3.36(13.90±3.10) | 8.98±3.84(14.80±5.30) |
| GA-7 | Nb-Methylgelsedilam | 16.73±4.50 | 13.65±6.47 | 9.98±5.53 |
| GA-9 | 19-Hydroxdihyogelsemine | 23.96±15.01 | 6.37±4.99 | 5.73±6.37 |
| GA-10 | 14-Hydroxygelsenicine | 3.86±5.45 | 16.61±4.22 | 6.04±3.99 |
| GA-11 | 11-Hydroxygelsenicine | 13.82±4.22 | 3.56±2.51 | 4.11±3.08 |
| GA-12 | Gelsevirine | 17.46±6.82 | 13.11±4.36 | 9.86±4.92 |
| GA-14 | Isomer of Akuammidine | 12.27±9.63 | 9.62±7.14 | 8.68±5.53 |
| GA-15 | Humantenine | 9.78±11.19 | 4.27±1.96 | 3.06±2.26 |
| GA-16 | Gelsemicine | 8.77±5.84 | 5.13±4.32 | 14.45±4.46 |
| GA-17 | Gelsemoxonine | 7.72±7.96 | 14.33±10.16 | 13.22±9.45 |
| GA-19 | Humantenoxenine | 6.40±8.19 | 16.11±11.27 | 12.73±11.11 |
| GA-20 | 15-Hydroxyhumantenine | 14.73±7.57 | 12.82±5.67 | 10.48±4.77 |
| GA-21 | 19-Hydroxydigelsevirine | 13.64±12.05 | 9.55±7.41 | 10.37±7.85 |
| GA-22 | 6-Hydroxyhumantenine | 3.72±18.27 | 15.46±6.40 | 15.42±6.39 |
| GA-23 | GS-2(11-Methoxy-14-Hydroxygelsenicine) | 7.16±3.48 | 10.58±8.25 | 10.81±4.29 |
| GA-24 | 14-Dehydrxoygelsefuranidine | 15.79±9.91 | 10.43±4.92 | 19.06±3.65 |
| GA-25 | Isomer of Dehydrxoygelsefuranidine(1) | 5.27±3.64 | 14.46±9.29 | 18.47±9.64 |
| GA-26 | Isomer of Dehydrxoygelsefuranidine(2) | 6.94±2.76 | 12.97±5.77 | 15.82±7.70 |
| GA-27 | 11-Methoxy-19-hydroxygelsegine | 15.07±10.36 | 5.14±5.52 | 6.83±6.96 |

*****: Represents the result of the precise quantitative method.

**Table S3.** The linearity results of two methods extrapolated to other pig tissues

| **Tissues** | **Compounds** | **Analytes** | **Standard curve** | **R^2^** | **Linear range (ng/mL)** |
| --- | --- | --- | --- | --- | --- |
| Heart | Koumidine | GA-1 | Y=175x+1.05e+003 | 0.9985 | 50-500 |
|  | Koumine | GA-2 | Y=163e+003x+1.32e+004(Y=281x+185) | 0.9951(0.9925) | 5-500(1-150) |
|  | Gelsemine | GA-4 | Y=1.03e+004x+1.02e+005(Y=1.6e+003x+2.06e+003) | 0.9922(0.9955) | 5-500(1-150) |
|  | Na-desmethoxyhumantenine | GA-5 | Y=323x+826 | 0.9974 | 50-500 |
|  | Gelsenicine | GA-6 | Y=1.31e+004x+1.27e+005(Y=2.72e+003x+7.73e+003) | 0.9917(0.9916) | 5-500(1-150) |
|  | Nb-Methylgelsedilam | GA-7 | Y=342x+3.55e+003 | 0.9953 | 20-500 |
|  | 19-Hydroxdihyogelsemine | GA-9 | Y=1.06e+003x+7.34e+003 | 0.9959 | 10-500 |
|  | 14-Hydroxygelsenicine | GA-10 | Y=2.12e+003x+6.41e+004 | 0.9958 | 5-500 |
|  | 11-Hydroxygelsenicine | GA-11 | Y=98.4x+2.88e+003 | 0.9905 | 20-500 |
|  | Gelsevirine | GA-12 | Y=1.52e+004x+8e+004 | 0.9934 | 5-500 |
|  | Isomer of Akuammidine | GA-14 | Y=1.42e+003x+5.42e+003 | 0.9933 | 5-500 |
|  | Humantenine | GA-15 | Y=1.2e+004x+9.84e+004 | 0.9942 | 5-500 |
|  | Gelsemicine | GA-16 | Y=5.94e+003x+3.63e+004 | 0.9939 | 5-500 |
|  | Gelsemoxonine | GA-17 | Y=125x+1.4e+003 | 0.9941 | 50-500 |
|  | Humantenoxenine | GA-19 | Y=1.78e+003x+1.04e+004 | 0.9928 | 10-500 |
|  | 15-Hydroxyhumantenine | GA-20 | Y=2.54e+003x+3.12e+004 | 0.9937 | 5-500 |
|  | 19-Hydroxydigelsevirine | GA-21 | Y=298x+3.29e+003 | 0.9939 | 20-500 |
|  | 6-Hydroxyhumantenine | GA-22 | Y=759x+6.66e+003 | 0.9943 | 5-500 |
|  | GS-2(11-Methoxy-14-Hydroxygelsenicine) | GA-23 | Y=179x+4.62e+003 | 0.9963 | 20-500 |
|  | 14-Dehydrxoygelsefuranidine | GA-24 | Y=440x+2.24e+003 | 0.9891 | 20-500 |
|  | Isomer of Dehydrxoygelsefuranidine(1) | GA-25 | Y=116x+955 | 0.9969 | 50-500 |
|  | Isomer of Dehydrxoygelsefuranidine(2) | GA-26 | Y=227x-768 | 0.9947 | 50-500 |
|  | 11-Methoxy-19-hydroxygelsegine | GA-27 | Y=293x+3.9e+003 | 0.9938 | 20-500 |
| Small intestine | Koumidine | GA-1 | Y=75.6x+1.03e+003 | 0.9688 | 5-500 |
|  | Koumine | GA-2 | Y=524x+3.09e+003(Y=253x+244) | 0.9985(0.9908) | 5-500(1-150) |
|  | Gelsemine | GA-4 | Y=3.01e+003x+3.77e+004(Y=964x+4.08e+003) | 0.9991(0.9903) | 5-500(1-150) |
|  | Na-desmethoxyhumantenine | GA-5 | Y=154x-724 | 0.9886 | 50-500 |
|  | Gelsenicine | GA-6 | Y=5.18e+003x+4.64e+004(Y=1.29e+004x+7.61e+003) | 0.9990(0.9967) | 5-500(1-150) |
|  | Nb-Methylgelsedilam | GA-7 | Y=123x+755 | 0.9978 | 20-500 |
|  | 19-Hydroxdihyogelsemine | GA-9 | Y=405x+5.48e+003 | 0.9958 | 10-500 |
|  | 14-Hydroxygelsenicine | GA-10 | Y=559x+1.44e+004 | 0.9914 | 5-500 |
|  | 11-Hydroxygelsenicine | GA-11 | Y=30x+5.29e+003 | 0.9895 | 20-500 |
|  | Gelsevirine | GA-12 | Y=4.97e+003x+2.05e+004 | 0.9975 | 5-500 |
|  | Isomer of Akuammidine | GA-14 | Y=535x+1.74e+003 | 0.9983 | 5-500 |
|  | Humantenine | GA-15 | Y=5.04e+003x+3.1e+004 | 0.9975 | 5-500 |
|  | Gelsemicine | GA-16 | Y=2.16e+003x+1.38e+004 | 0.9960 | 5-500 |
|  | Gelsemoxonine | GA-17 | Y=32.3x+935 | 0.9849 | 50-500 |
|  | Humantenoxenine | GA-19 | Y=598x+912 | 0.9945 | 10-500 |
|  | 15-Hydroxyhumantenine | GA-20 | Y=960x+2.28e+004 | 0.9860 | 5-500 |
|  | 19-Hydroxydigelsevirine | GA-21 | Y=81.7x+610 | 0.9991 | 20-500 |
|  | 6-Hydroxyhumantenine | GA-22 | Y=276x+2.67e+003 | 0.9959 | 5-500 |
|  | GS-2(11-Methoxy-14-Hydroxygelsenicine) | GA-23 | Y=44.9x+332 | 0.9994 | 20-500 |
|  | 14-Dehydrxoygelsefuranidine | GA-24 | Y=212x-29.5 | 0.9976 | 20-500 |
|  | Isomer of Dehydrxoygelsefuranidine(1) | GA-25 | Y=55.3x+903 | 0.9851 | 50-500 |
|  | Isomer of Dehydrxoygelsefuranidine(2) | GA-26 | Y=111x-467 | 0.9969 | 50-500 |
|  | 11-Methoxy-19-hydroxygelsegine | GA-27 | Y=129x+1.07e+003 | 0.9929 | 20-500 |
| Spleen | Koumidine | GA-1 | Y=125x+122 | 0.9902 | 50-500 |
|  | Koumine | GA-2 | Y=1.39e+003x+8.02e+003(Y=497x+464) | 0.9861(0.9928) | 5-500(1-150) |
|  | Gelsemine | GA-4 | Y=9.22e+003x+5.53e+004(Y=2.04e+003x+3.31e+003) | 0.9926(0.9938) | 5-500(1-150) |
|  | Na-desmethoxyhumantenine | GA-5 | Y=178x+3.38e+003 | 0.9928 | 50-500 |
|  | Gelsenicine | GA-6 | Y=7.43e+003x+1e+005(Y=2.91e+004x+1.56e+004) | 0.9786(0.9903) | 5-500(1-150) |
|  | Nb-Methylgelsedilam | GA-7 | Y=225x+821 | 0.9860 | 20-500 |
|  | 19-Hydroxdihyogelsemine | GA-9 | Y=462x+2.16e+004 | 0.9916 | 10-500 |
|  | 14-Hydroxygelsenicine | GA-10 | Y=1.4e+003x+4.71e+004 | 0.9935 | 5-500 |
|  | 11-Hydroxygelsenicine | GA-11 | Y=73.8x+5.77e+003 | 0.9862 | 20-500 |
|  | Gelsevirine | GA-12 | Y=8.25e+003x+3.37e+004 | 0.9906 | 5-500 |
|  | Isomer of Akuammidine | GA-14 | Y=1.03e+003x+3.04e+003 | 0.9949 | 5-500 |
|  | Humantenine | GA-15 | Y=7.72e+003x+5.82e+004 | 0.9854 | 5-500 |
|  | Gelsemicine | GA-16 | Y=3.48e+003x+1.76e+004 | 0.9901 | 5-500 |
|  | Gelsemoxonine | GA-17 | Y=77.1x+3.37e+003 | 0.9955 | 50-500 |
|  | Humantenoxenine | GA-19 | Y=1.06e+003x+7.11e+003 | 0.9921 | 10-500 |
|  | 15-Hydroxyhumantenine | GA-20 | Y=1.29e+003x+3.43e+004 | 0.9936 | 5-500 |
|  | 19-Hydroxydigelsevirine | GA-21 | Y=184x+1.05e+003 | 0.9869 | 20-500 |
|  | 6-Hydroxyhumantenine | GA-22 | Y=373x+5.54e+003 | 0.9962 | 5-500 |
|  | GS-2(11-Methoxy-14-Hydroxygelsenicine) | GA-23 | Y=109x+1.02e+003 | 0.9877 | 20-500 |
|  | 14-Dehydrxoygelsefuranidine | GA-24 | Y=268x+585 | 0.9891 | 20-500 |
|  | Isomer of Dehydrxoygelsefuranidine(1) | GA-25 | Y=75.2x+126 | 0.9831 | 50-500 |
|  | Isomer of Dehydrxoygelsefuranidine(2) | GA-26 | Y=143x-157 | 0.9824 | 50-500 |
|  | 11-Methoxy-19-hydroxygelsegine | GA-27 | Y=150x+1.89e+003 | 0.9820 | 20-500 |
| Urine | Koumidine | GA-1 | Y=2.26e+003x+8.33e+003 | 0.9971 | 10-200 |
|  | Koumine | GA-2 | Y=3.09e+003x-1.65e+003(Y=1.04e+003x+1.7e+003) | 0.9985(0.9943) | 5-200(0.2-200) |
|  | 3-Hydroxykoumidine | GA-3 | Y=346x+742 | 0.9997 | 20-200 |
|  | Gelsemine | GA-4 | Y=1.83e+004x+6.6e+003(Y=2.43e+003x+9.8e+003) | 0.9995(0.9986) | 1-200(0.2-200) |
|  | Na-desmethoxyhumantenine | GA-5 | Y=587x-678 | 0.9997 | 20-200 |
|  | Gelsenicine | GA-6 | Y=2.67e+004x+8.18e+003(Y=1.43e+003x+4.6e+003) | 0.9998(0.9970) | 1-200(0.2-200) |
|  | Nb-Methylgelsedilam | GA-7 | Y=680x-1.46e+003 | 0.9991 | 20-200 |
|  | 19-Hydroxdihyogelsemine | GA-9 | Y=1.63e+003x-83.8 | 0.9992 | 5-200 |
|  | 14-Hydroxygelsenicine | GA-10 | Y=472x+414 | 0.9995 | 10-200 |
|  | 11-Hydroxygelsenicine | GA-11 | Y=611x+497 | 0.9996 | 20-200 |
|  | Gelsevirine | GA-12 | Y=2.22e+004x+333 | 0.9994 | 1-200 |
|  | 19-Akuammidine | GA-13 | Y=856x-298 | 0.9978 | 10-200 |
|  | Isomer of Akuammidine | GA-14 | Y=3.01e+003x-2.14e+003 | 0.9990 | 10-200 |
|  | Humantenine | GA-15 | Y=1.91e+004x+1.81e+003 | 0.9998 | 1-200 |
|  | Gelsemicine | GA-16 | Y=6.03e+003x+1.36e+003 | 0.9998 | 1-200 |
|  | Gelsemoxonine | GA-17 | Y=174x-141 | 0.9994 | 20-200 |
|  | Humantenoxenine | GA-19 | Y=2.23e+003x-2.43e+003 | 0.9932 | 5-200 |
|  | 15-Hydroxyhumantenine | GA-20 | Y=4.45e+003x+109 | 0.9990 | 5-200 |
|  | 19-Hydroxydigelsevirine | GA-21 | Y=569x-976 | 0.9992 | 20-200 |
|  | 6-Hydroxyhumantenine | GA-22 | Y=1.24e+003x-587 | 0.9978 | 5-200 |
|  | GS-2(11-Methoxy-14-Hydroxygelsenicine) | GA-23 | Y=295x-152 | 0.9997 | 20-200 |
|  | 14-Dehydrxoygelsefuranidine | GA-24 | Y=694x-153 | 0.9996 | 10-200 |
|  | Isomer of Dehydrxoygelsefuranidine(1) | GA-25 | Y=196x-359 | 0.9988 | 20-200 |
|  | 11-Methoxy-19-hydroxygelsegine | GA-27 | Y=612x+200 | 0.9987 | 10-200 |
| Brain | Koumidine | GA-1 | Y=63.9x-125 | 0.9810 | 50-500 |
|  | Koumine | GA-2 | Y=889x+1.5e+003(Y=396+239) | 0.9985(0.9992) | 5-500(1-150) |
|  | Gelsemine | GA-4 | Y=5.01e+003x+4.13e+004(Y=1.3e+003x+2.06e+003) | 0.9896(0.9937) | 5-500(1-150) |
|  | Na-desmethoxyhumantenine | GA-5 | Y=115x+415 | 0.9859 | 50-500 |
|  | Gelsenicine | GA-6 | Y=6.51e+003x+2.8e+004(Y=2.34e+004x+5.43e+003) | 0.9950(0.9995) | 5-500(1-150) |
|  | Nb-Methylgelsedilam | GA-7 | Y=138x+1.21e+003 | 0.9948 | 20-500 |
|  | 19-Hydroxdihyogelsemine | GA-9 | Y=413x+2.75e+003 | 0.9931 | 10-500 |
|  | 14-Hydroxygelsenicine | GA-10 | Y=761x+1.83e+004 | 0.9825 | 5-500 |
|  | 11-Hydroxygelsenicine | GA-11 | Y=53.5x+1.18e+003 | 0.9823 | 20-500 |
|  | Gelsevirine | GA-12 | Y=5.98e+003x+1.09e+004 | 0.9993 | 5-500 |
|  | Isomer of Akuammidine | GA-14 | Y=515x+1.46e+003 | 0.9896 | 5-500 |
|  | Humantenine | GA-15 | Y=5.41e+003x+2.32e+004 | 0.9992 | 5-500 |
|  | Gelsemicine | GA-16 | Y=1.98e+003x+6.45e+003 | 0.9831 | 5-500 |
|  | Gelsemoxonine | GA-17 | Y=52.3x+1.09e+003 | 0.9937 | 50-500 |
|  | Humantenoxenine | GA-19 | Y=620x-415 | 0.9937 | 10-500 |
|  | 15-Hydroxyhumantenine | GA-20 | Y=827x+2.88e+003 | 0.9969 | 5-500 |
|  | 19-Hydroxydigelsevirine | GA-21 | Y=101x-144 | 0.9963 | 20-500 |
|  | 6-Hydroxyhumantenine | GA-22 | Y=250x+244 | 0.9976 | 5-500 |
|  | GS-2(11-Methoxy-14-Hydroxygelsenicine) | GA-23 | Y=72.7x+18 | 0.9947 | 20-500 |
|  | 14-Dehydrxoygelsefuranidine | GA-24 | Y=201x-866 | 0.9971 | 20-500 |
|  | Isomer of Dehydrxoygelsefuranidine(1) | GA-25 | Y=64.9x+32.8 | 0.9999 | 50-500 |
|  | Isomer of Dehydrxoygelsefuranidine(2) | GA-26 | Y=114x+149 | 0.9995 | 50-500 |
|  | 11-Methoxy-19-hydroxygelsegine | GA-27 | Y=125x-429 | 0.9916 | 20-500 |
| Spinal cord | Koumidine | GA-1 | Y=146x+9.45e+003 | 0.9934 | 50-500 |
|  | Koumine | GA-2 | Y=403x+470(Y=567+335) | 0.9952(0.9924) | 5-500(1-150) |
|  | Gelsemine | GA-4 | Y=1.72e+003x+3.13e+004(Y=1.81e+003x+2.11e+003) | 0.9974(0.9984) | 5-500(1-150) |
|  | Na-desmethoxyhumantenine | GA-5 | Y=92.8x-559 | 0.9836 | 50-500 |
|  | Gelsenicine | GA-6 | Y=2.99e+003x+1.33e+004(Y=2.96e+004x+2.46e+003) | 0.9935(0.9987) | 5-500(1-150) |
|  | Nb-Methylgelsedilam | GA-7 | Y=70.9x+4.36e+003 | 0.9903 | 20-500 |
|  | 19-Hydroxdihyogelsemine | GA-9 | Y=313x+1.55e+003 | 0.9911 | 10-500 |
|  | 14-Hydroxygelsenicine | GA-10 | Y=285x+5.92e+003 | 0.9941 | 5-500 |
|  | 11-Hydroxygelsenicine | GA-11 | Y=40.8x+516 | 0.9949 | 20-500 |
|  | Gelsevirine | GA-12 | Y=2.81e+003x+3.8e+003 | 0.9946 | 5-500 |
|  | Isomer of Akuammidine | GA-14 | Y=276x+1.53e+003 | 0.9934 | 5-500 |
|  | Humantenine | GA-15 | Y=3.35e+003x+7.37e+003 | 0.9935 | 5-500 |
|  | Gelsemicine | GA-16 | Y=1.52e+003x+3.84e+003 | 0.9972 | 5-500 |
|  | Gelsemoxonine | GA-17 | Y=13.2x+935 | 0.9981 | 50-500 |
|  | Humantenoxenine | GA-19 | Y=296x+1.1e+003 | 0.9926 | 10-500 |
|  | 15-Hydroxyhumantenine | GA-20 | Y=524x+7.32e+003 | 0.9913 | 5-500 |
|  | 19-Hydroxydigelsevirine | GA-21 | Y=48.1x+154 | 0.9926 | 20-500 |
|  | 6-Hydroxyhumantenine | GA-22 | Y=193x+1.54e+003 | 0.9963 | 5-500 |
|  | GS-2(11-Methoxy-14-Hydroxygelsenicine) | GA-23 | Y=23x+893 | 0.9940 | 20-500 |
|  | 14-Dehydrxoygelsefuranidine | GA-24 | Y=136x+340 | 0.9988 | 20-500 |
|  | Isomer of Dehydrxoygelsefuranidine(1) | GA-25 | Y=37.5x+45.3 | 0.9941 | 50-500 |
|  | Isomer of Dehydrxoygelsefuranidine(2) | GA-26 | Y=66.1x+51.5 | 0.9994 | 50-500 |
|  | 11-Methoxy-19-hydroxygelsegine | GA-27 | Y=71.2x+540 | 0.9987 | 20-500 |
| Lungs | Koumidine | GA-1 | Y=91x+4.66e+003 | 0.9564 | 50-500 |
|  | Koumine | GA-2 | Y=1.41e+003x+8.88e+003(Y=506x+878) | 0.9959(0.9915) | 5-500(1-150) |
|  | Gelsemine | GA-4 | Y=8.36e+003x+6.06e+004(1.83e+003x+6.6e+003) | 0.9882(0.9972) | 5-500(1-150) |
|  | Na-desmethoxyhumantenine | GA-5 | Y=199x+3.49e+003 | 0.9980 | 50-500 |
|  | Gelsenicine | GA-6 | Y=9.83e+003x+6.61e+004(2.86e+004x+2.75e+004) | 0.9978(0.9915) | 5-500(1-150) |
|  | Nb-Methylgelsedilam | GA-7 | Y=239x+1.88e+003 | 0.9954 | 20-500 |
|  | 19-Hydroxdihyogelsemine | GA-9 | Y=609x+1.46e+004 | 0.9909 | 10-500 |
|  | 14-Hydroxygelsenicine | GA-10 | Y=1.3e+003x+4.27e+004 | 0.9920 | 5-500 |
|  | 11-Hydroxygelsenicine | GA-11 | Y=64.6x+4.11e+003 | 0.9851 | 20-500 |
|  | Gelsevirine | GA-12 | Y=9.44e+003x+4.38e+004 | 0.9986 | 5-500 |
|  | Isomer of Akuammidine | GA-14 | Y=1.01e+003x+3.09e+003 | 0.9916 | 5-500 |
|  | Humantenine | GA-15 | Y=8.06e+003x+4.94e+004 | 0.9967 | 5-500 |
|  | Gelsemicine | GA-16 | Y=3.24e+003x+4.38e+004 | 0.9909 | 5-500 |
|  | Gelsemoxonine | GA-17 | Y=67.9x+3.41e+003 | 0.9911 | 50-500 |
|  | Humantenoxenine | GA-19 | Y=1.07e+003x+7.12e+003 | 0.9989 | 10-500 |
|  | 15-Hydroxyhumantenine | GA-20 | Y=1.32e+003x+2.53e+004 | 0.9950 | 5-500 |
|  | 19-Hydroxydigelsevirine | GA-21 | Y=157x+3.3e+003 | 0.9904 | 20-500 |
|  | 6-Hydroxyhumantenine | GA-22 | Y=352x+5.44e+003 | 0.9929 | 5-500 |
|  | GS-2(11-Methoxy-14-Hydroxygelsenicine) | GA-23 | Y=104x+1.31e+003 | 0.9924 | 20-500 |
|  | 14-Dehydrxoygelsefuranidine | GA-24 | Y=249x+2.2e+003 | 0.9915 | 20-500 |
|  | Isomer of Dehydrxoygelsefuranidine(1) | GA-25 | Y=77.3x+1.02e+003 | 0.9808 | 50-500 |
|  | Isomer of Dehydrxoygelsefuranidine(2) | GA-26 | Y=128x+1.65e+003 | 0.9940 | 50-500 |
|  | 11-Methoxy-19-hydroxygelsegine | GA-27 | Y=170x+3.71e+003 | 0.9814 | 20-500 |
| Bile | Koumidine | GA-1 | Y=1.39e+003x+5.65e+004 | 0.9955 | 5-200 |
|  | Koumine | GA-2 | Y=1.68e+003x+5.46e+003(8.52e+003x+4.3e+003) | 0.9998(0.9946) | 20-200(0.2-200) |
|  | 3-Hydroxykoumidine | GA-3 | Y=241x+5.68e+003 | 0.9997 | 1-200 |
|  | Gelsemine | GA-4 | Y=1.2e+004x+8.14e+004(4.53e+004x+2.8e+004) | 0.9962(0.9973) | 20-200(0.2-200) |
|  | Na-desmethoxyhumantenine | GA-5 | Y=458x+2.32e+003 | 0.9997 | 1-200 |
|  | Gelsenicine | GA-6 | Y=1.8e+004x+4.2e+004(3.43e+004x+5.53e+003) | 0.9984(0.9972) | 20-200(0.2-200) |
|  | Nb-Methylgelsedilam | GA-7 | Y=418x+2.2e+003 | 1 | 5-200 |
|  | 19-Hydroxdihyogelsemine | GA-9 | Y=1.38e+003x+7.44e+003 | 0.9995 | 10-200 |
|  | 14-Hydroxygelsenicine | GA-10 | Y=635x+2.34e+003 | 0.9994 | 20-200 |
|  | 11-Hydroxygelsenicine | GA-11 | Y=286x+7.46e+003 | 0.9984 | 1-200 |
|  | Gelsevirine | GA-12 | Y=1.32e+004x+4.12e+004 | 0.9996 | 10-200 |
|  | Isomer of Akuammidine | GA-14 | Y=1.66e+003x+51.2 | 0.9972 | 1-200 |
|  | Gelsemicine | GA-16 | Y=1.02e+004x+133 | 0.9854 | 5-200 |
|  | Gelselegine | GA-18 | Y=2.57e+003x+31 | 0.9807 | 5-200 |
|  | Humantenoxenine | GA-19 | Y=2.51e+003x+133 | 0.9979 | 5-200 |
|  | 15-Hydroxyhumantenine | GA-20 | Y=3.57e+003x+38.8 | 0.9976 | 20-200 |
|  | 19-Hydroxydigelsevirine | GA-21 | Y=382x+325 | 0.9927 | 5-200 |
|  | 6-Hydroxyhumantenine | GA-22 | Y=1.13e+003x+45.3 | 0.9981 | 20-200 |
|  | GS-2(11-Methoxy-14-Hydroxygelsenicine) | GA-23 | Y=245x+ e-012 | 0.9991 | 10-200 |
|  | 14-Dehydrxoygelsefuranidine | GA-24 | Y=821x+14.8 | 0.9996 | 20-200 |
|  | Isomer of Dehydrxoygelsefuranidine(1) | GA-25 | Y=70.1x+16.1 | 0.9971 | 20-200 |
|  | Isomer of Dehydrxoygelsefuranidine(2) | GA-26 | Y=316x+25.1 | 0.9999 | 20-200 |
|  | 11-Methoxy-19-hydroxygelsegine | GA-27 | Y=631x+42.8 | 0.9825 | 20-200 |

**CAPTIONS FOR FIGURES**

**Fig S1.** Representative MRM chromatograms of three alkaloids by the precise quantitation method: (A) Blank pig tissues sample (muscle: gelemine-a1, koumine-a2 and gelsenicine-a3; liver: gelsemine-b1, koumine-b2 and gelsenicine-b3; kidney: gelsemine-c1, koumine-c2 and gelsenicine-c3) and (B) blank pig tissue sample spiked with the three alkaloids (muscle: gelemine-d1, koumine-d2 and gelsenicine-d3; liver: gelsemine-e1, koumine-e2 and gelsenicine-e3; kidney: gelsemine-f1, koumine-f2 and gelsenicine-f3).

**Fig S2.** The representative sMRM chromatograms of 22 alkaloids by the semiquantitative method: (A) Blank pig muscle tissue sample spiked with Gelsemium total alkaloids and (B) blank pig muscle tissue sample.

**Fig S3.** The representative sMRM chromatograms of 22 alkaloids by the semiquantitative method: (A) Blank pig liver tissue sample spiked with Gelsemium total alkaloids and (B) blank pig liver tissue sample.

**Fig S4.** The representative sMRM chromatograms of 22 alkaloids by the semiquantitative method: (A) Blank pig kidney tissue sample spiked with Gelsemium total alkaloids and (B) blank pig kidney tissue sample.

**Fig S5.** Performance and quality control of the strategy based method for 22 alkaloids and heat maps showing the accuracy and interday precision of each alkaloid in quality control samples in 3 different concentrations ranges: low, 5.0, 10.0, 20.0 and 50.0 μg/L; medium, 50.0 and 100.0 μg/L; and high, 100.0 and 150.0 μg/L; (A) muscle; (B) liver and (C) kidney.

**Fig S6.** The correlation coefficients of 14-hydroxygelsenicine between the concentration in the (A) liver, (B) kidneys, (C) intestine and (D) pancreas tissues and that in the urine; the correlation coefficients of gelsemoxonine between the concentration in the (E) liver and (F) pancreas tissues and that in the urine; the correlation coefficients of gelsemoxonine between the concentration in the (G) liver and (H) pancreas and that in the plasma.


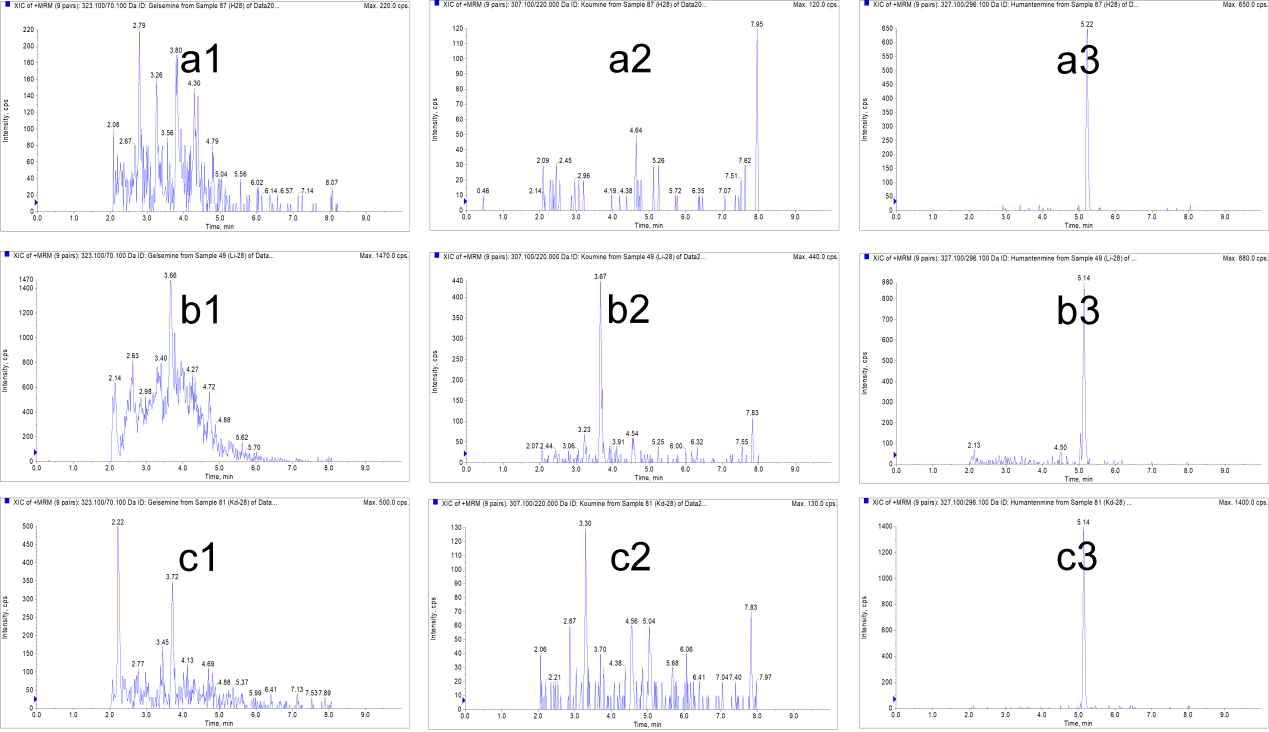


**Fig S1 A**


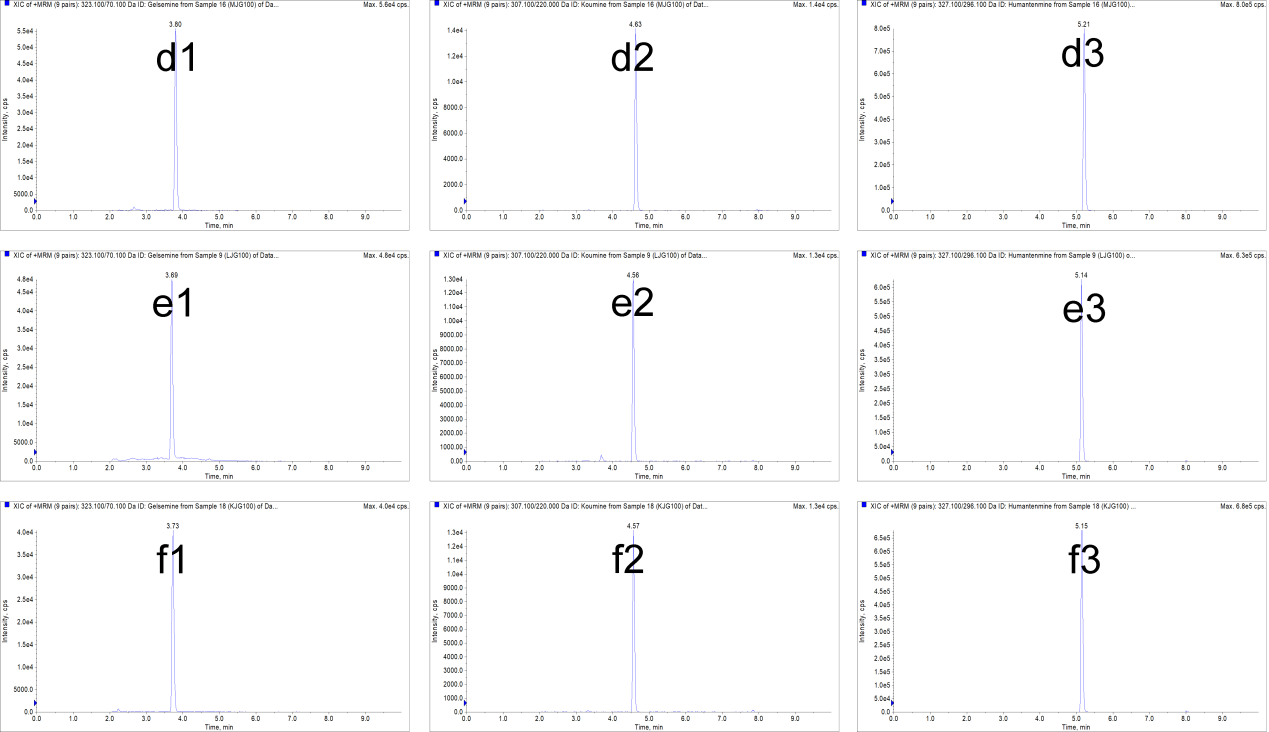


**Fig S1 B**


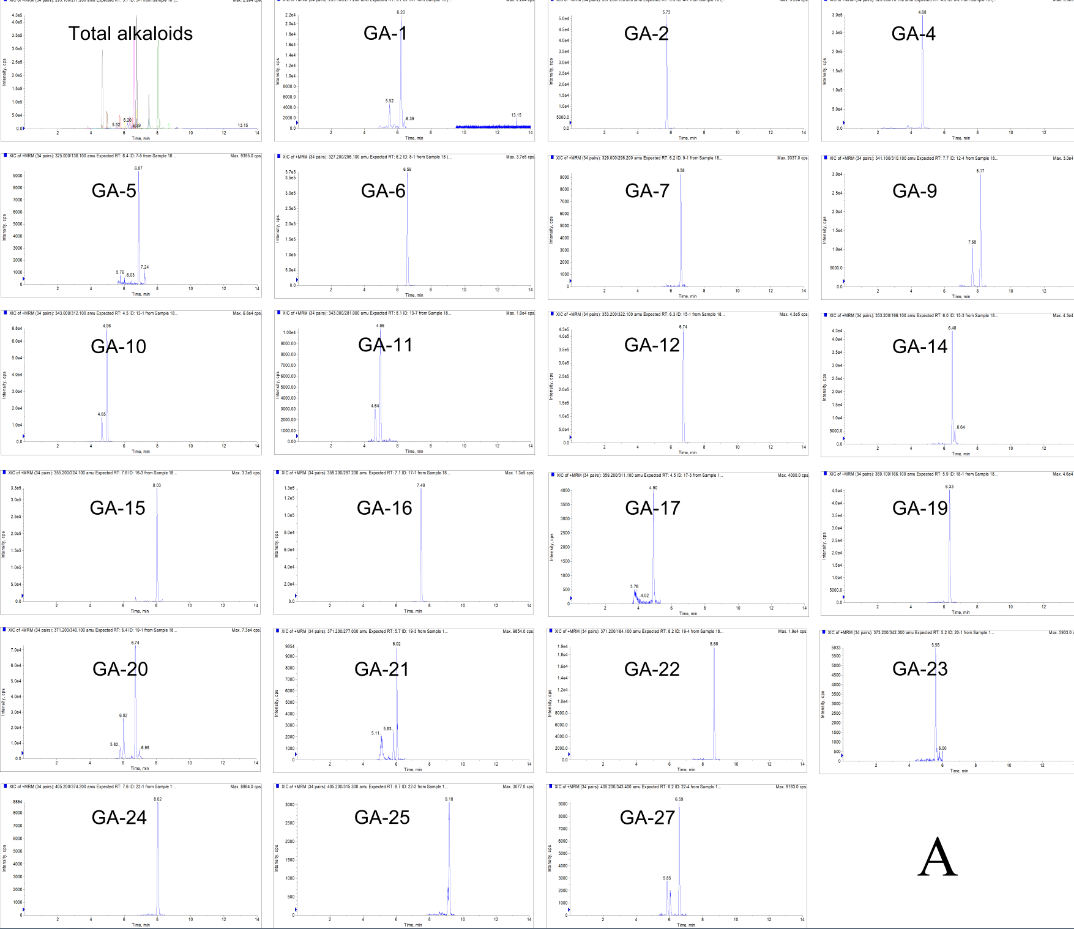


**Fig S2 A**


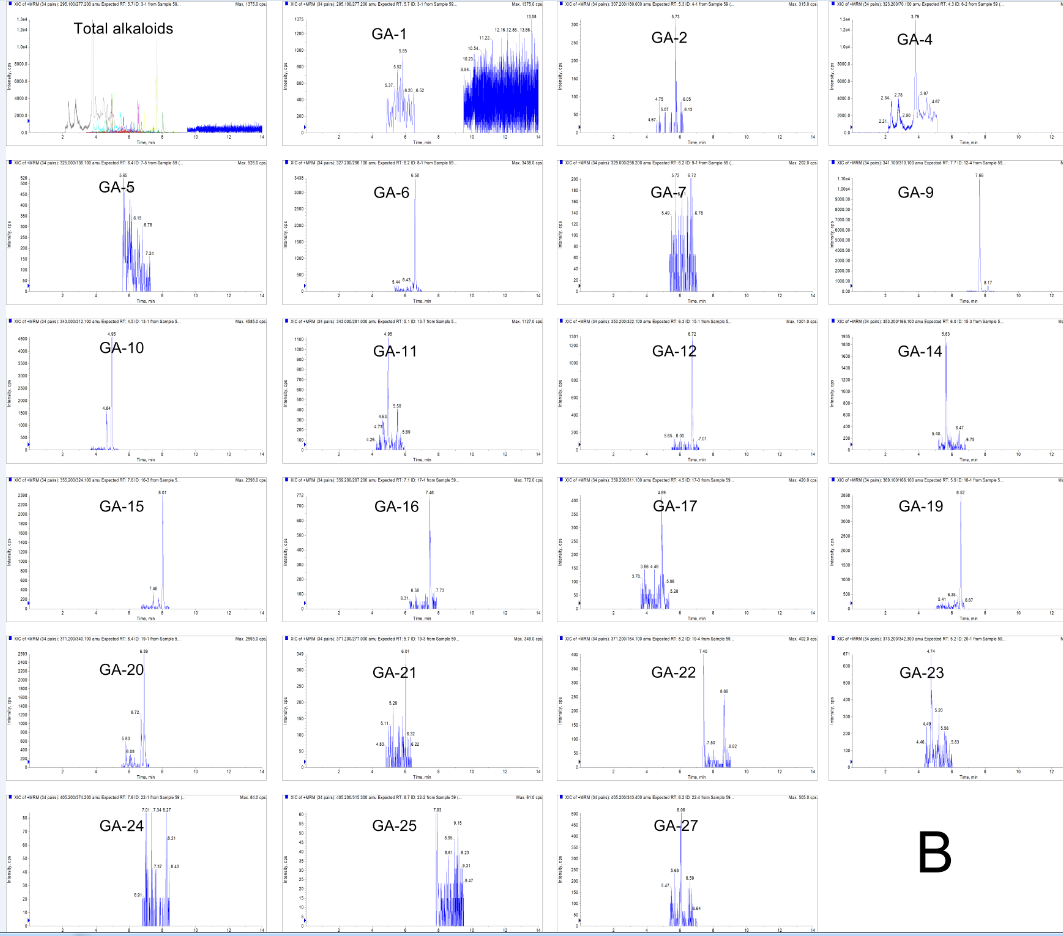


**Fig S2 B**


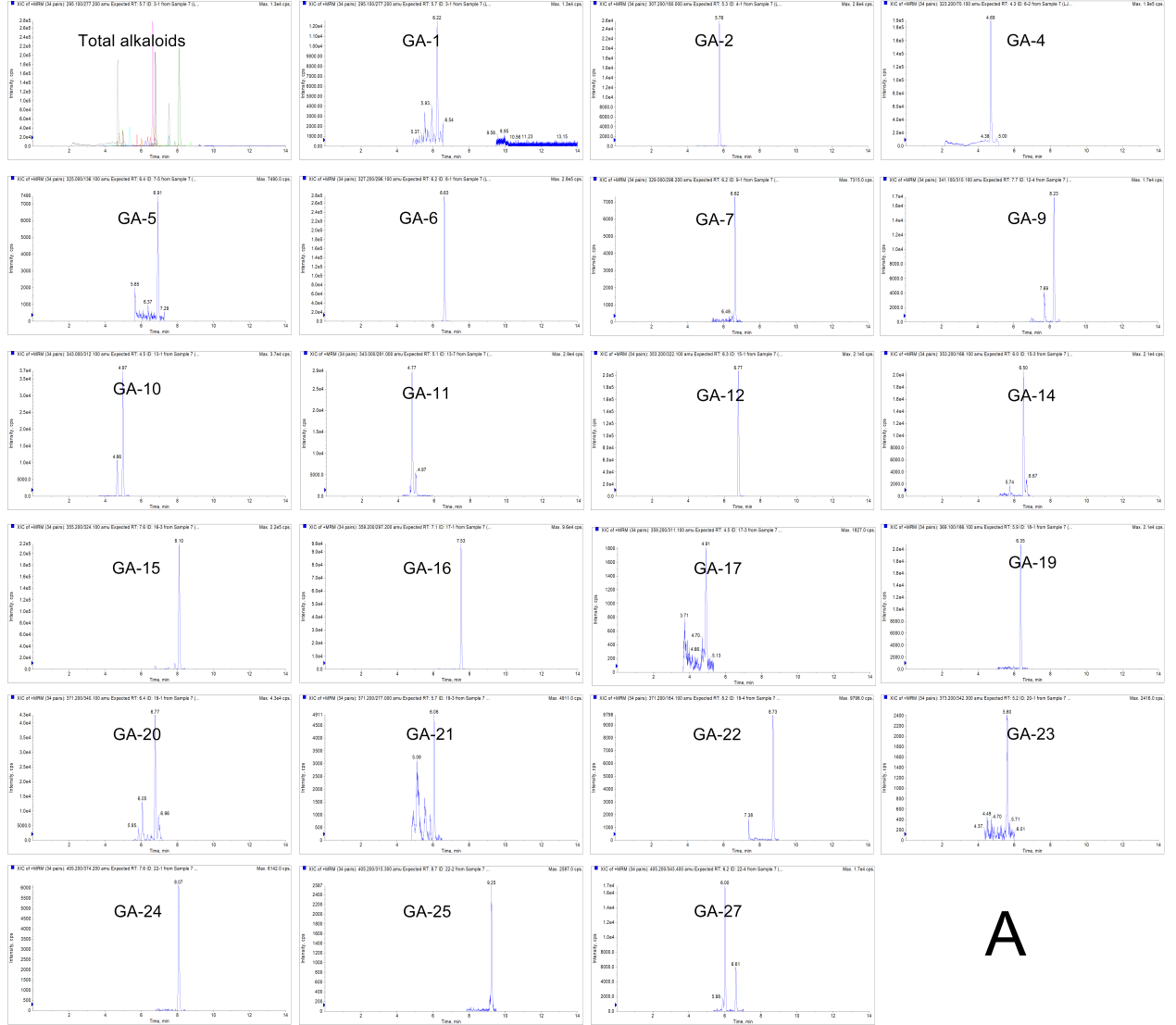


**Fig S3 A**


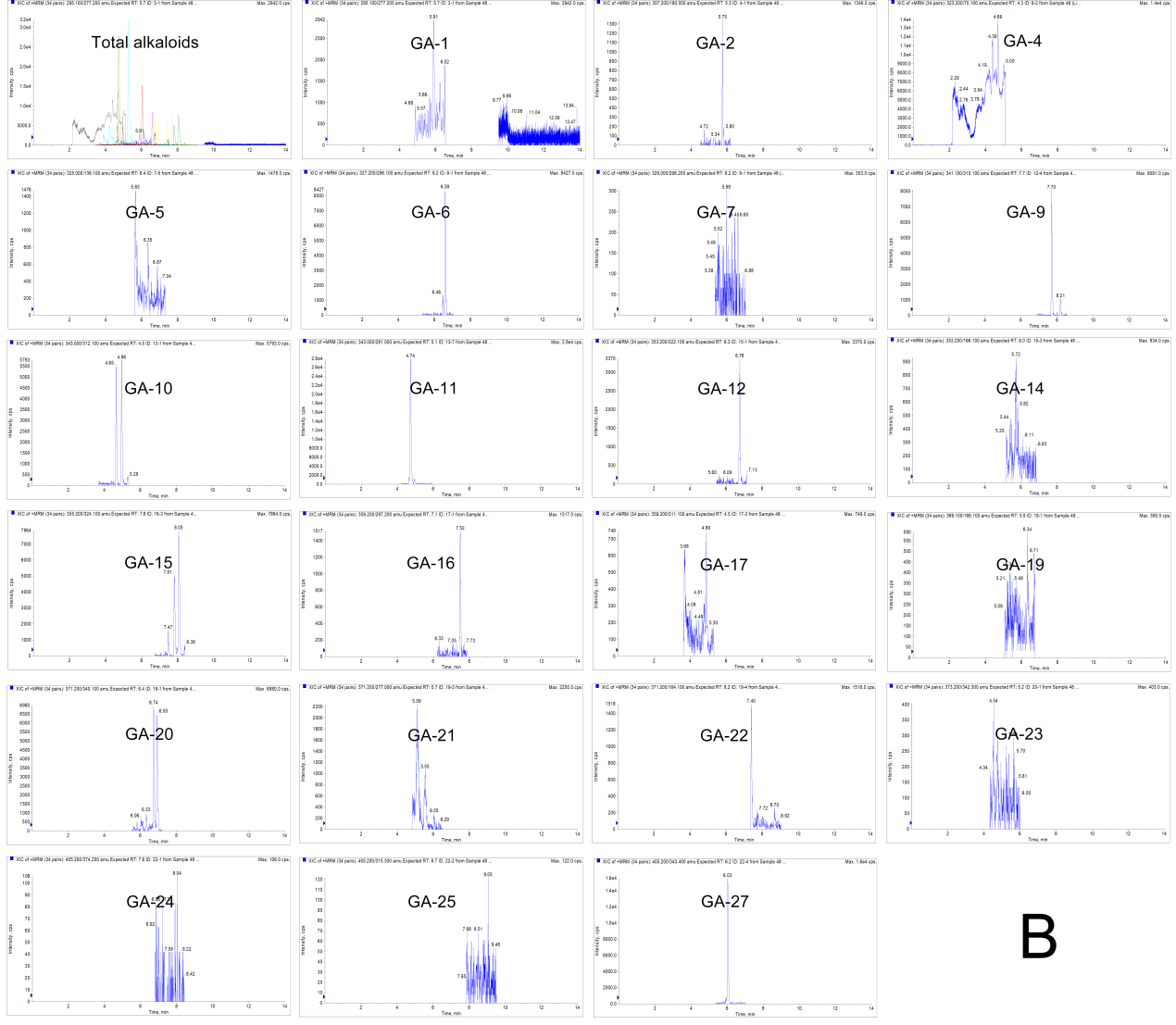


**Fig S3 B**


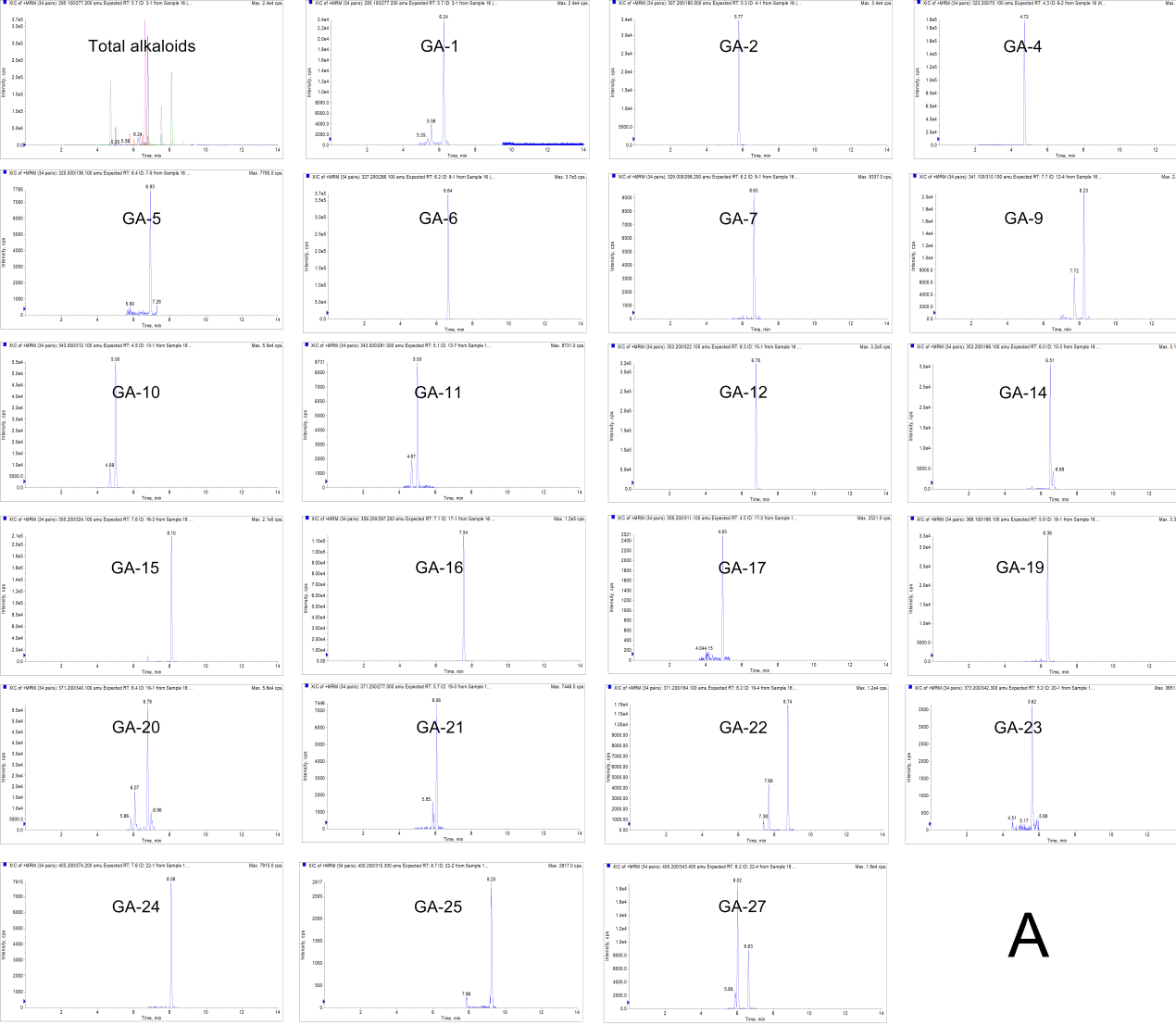


**Fig S4 A**


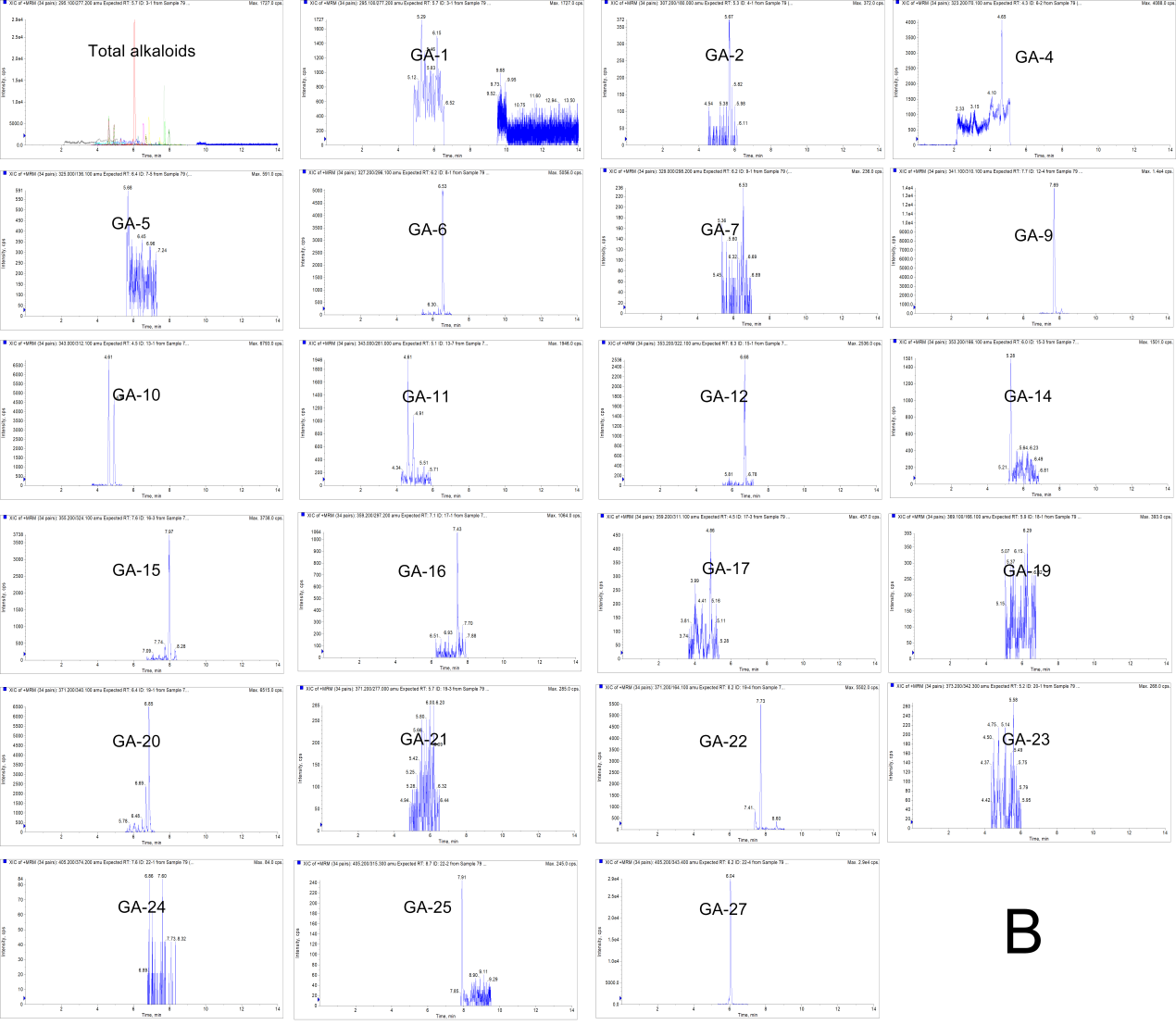


**Fig S4 B**


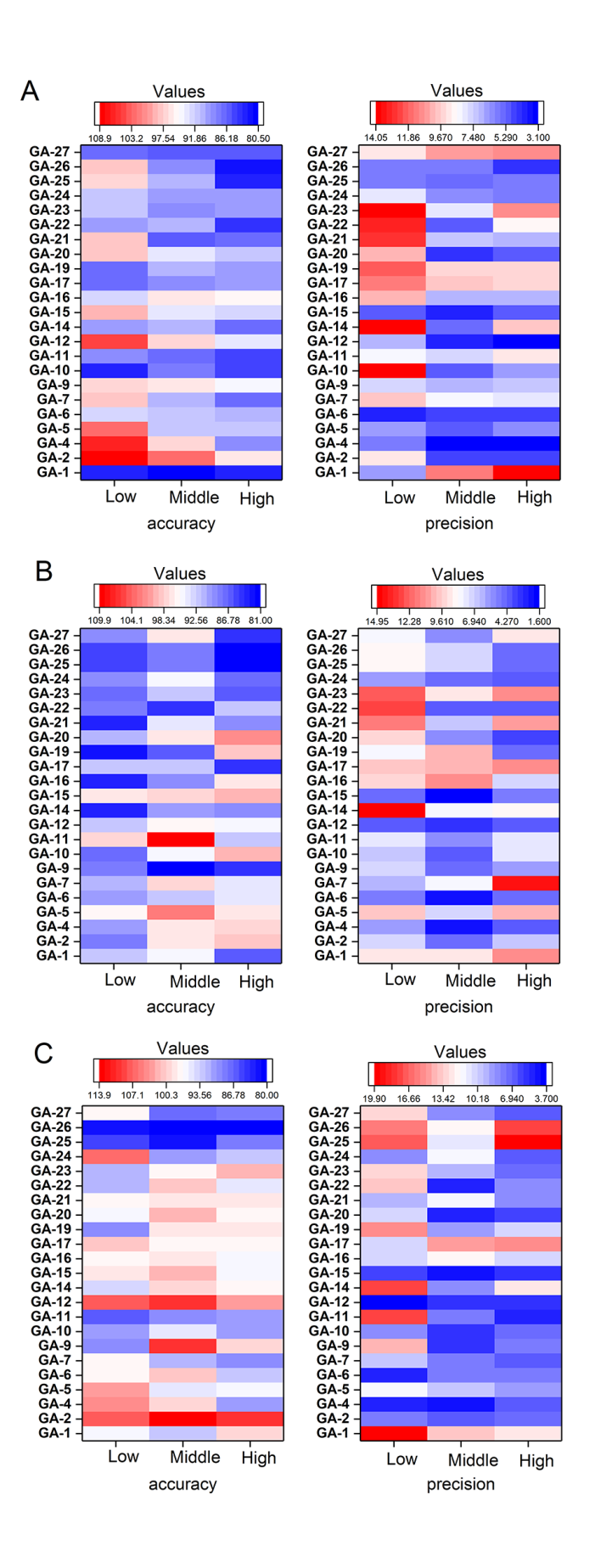


**Fig S5**





**Fig S6**
